# Transcriptional and epigenetic signatures of zygotic genome activation during early drosophila embryogenesis

**DOI:** 10.1186/1471-2164-14-226

**Published:** 2013-04-05

**Authors:** Elodie Darbo, Carl Herrmann, Thomas Lecuit, Denis Thieffry, Jacques van Helden

**Affiliations:** 1 Technological Advances for Genomics and Clinics (TAGC), INSERM U1090, Université de la Méditerranée, Campus de Luminy, 13288 Marseille Cedex 9, France; 2, Institut de Biologie du Développement de Marseille-Luminy (IBDML), UMR 7288 Case 907 - Parc Scientifique de Luminy, 13288 Marseille Cedex 9, France; 3, Institut de Biologie de l’Ecole Normale Supérieure (IBENS) - UMR ENS and CNRS 8197 and INSERM 1024, 46 rue d’Ulm, 75005 Paris, France; 4 Laboratoire de Bioinformatique des Génomes et des Réseaux (BiGRe), Université Libre de Bruxelles, Campus Plaine, CP 263, Bld du Triomphe, B-1050 Bruxelles, Belgium

**Keywords:** Drosophila Melanogaster, Zygotic Genome Activation, Transcriptional Regulation, Epigenetic Regulation, Transcriptome, ChIP-seq

## Abstract

**Background:**

In all Metazoa, transcription is inactive during the first mitotic cycles after fertilisation. In *Drosophila melanogaster*, Zygotic Genome Activation (ZGA) occurs in two waves, starting respectively at mitotic cycles 8 (approximately 60 genes) and 14 (over a thousand genes). The regulatory mechanisms underlying these drastic transcriptional changes remain largely unknown.

**Results:**

We developed an original gene clustering method based on discretized transition profiles, and applied it to datasets from three landmark early embryonic transcriptome studies. We identified 417 genes significantly up-regulated during ZGA. *De novo* motif discovery returned nine motifs over-represented in their non-coding sequences (upstream, introns, UTR), three of which correspond to previously known transcription factors: Zelda, Tramtrack and Trithorax-like (Trl). The nine discovered motifs were combined to scan ZGA-associated regions and predict about 1300 putative cis-regulatory modules. The fact that Trl is known to act as chromatin remodelling factor suggests that epigenetic regulation might play an important role in zygotic genome activation. We thus systematically compared the locations of predicted CRMs with ChIP-seq profiles for various transcription factors, 38 epigenetic marks from ModENCODE, and DNAse1 accessibility profiles. This analysis highlighted a strong and specific enrichment of predicted ZGA-associated CRMs for Zelda, CBP, Trl binding sites, as well as for histone marks associated with active enhancers (H3K4me1) and for open chromatin regions.

**Conclusion:**

Based on the results of our computational analyses, we suggest a temporal model explaining the onset of zygotic genome activation by the combined action of transcription factors and epigenetic signals. Although this study is mainly based on the analysis of publicly available transcriptome and ChiP-seq datasets, the resulting model suggests novel mechanisms that underly the coordinated activation of several hundreds genes at a precise time point during embryonic development.

## Background

**Figure 1:**
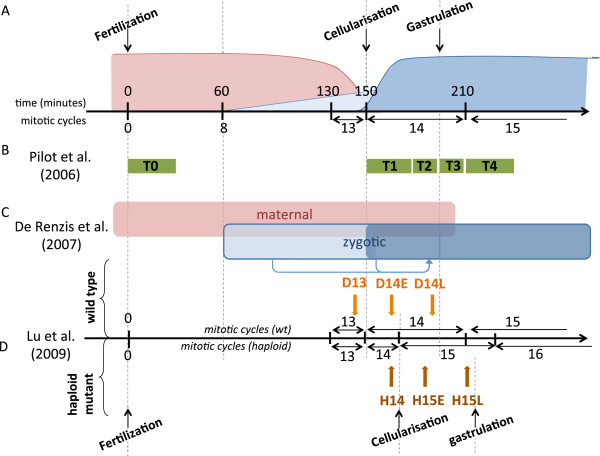
**Temporal organization of Zygotic Genome Activation and design of published high-throughput experiments used to extract early induced gene clusters.(A)** Schematic representation of mRNA concentration evolution during early drosophila embryogenesis. The horizontal axis represents time, in minutes after fertilisation (upper scale) or mitotic cycles (lower scale). Red: maternal mRNAs; light blue: first wave of zygotic transcription; dark blue: second wave of zygotic transcription. **(B)** Time points sampled in transcriptome microarray experiments performed by Pilot et al. (2006). **(C)** Respective contribution of maternal and zygotic mRNAs. Arrows represent the action of early expressed TF on secondary targets. **(D)** Time points of transcriptome microarray experiments published by Lu et al. (2009). D and H denote diploids and haploids; the numbers indicate the mitotic cycle number; E and L stand for early and late. Developmental milestones are indicated for haploid mutant embryos; notice the differences with wild-type timing in **(A)**.

During the earliest stages of development, metazoan embryos undergo drastic morphological changes and transcriptional reprogramming. Just after fertilisation, while the zygotic genome is transcriptionally inactive, developmental control is ensured by maternal products (mRNAs and proteins) loaded in the egg during oogenesis. After a species-dependent number of mitotic cycles, the zygotic genome is activated and takes control of embryonic development, whereas maternal mRNAs are actively degraded. Known as the “maternal-to-zygotic transition” (MZT), this fundamental process is conserved between metazoans [1]. Zygotic Genome Activation (ZGA) occurs in two successive waves: a minor wave involving a few tens of genes, followed by a major wave affecting several hundreds of genes (Figure 1A).

After fertilisation, *Drosophila melanogaster* embryos undergo a series of 13 fast mitotic divisions without cytokinesis (thus forming a syncytium, i.e. a single cell with multiple nuclei). The first seven mitotic cycles are fast (8 min/cycle) and synchronous, while the zygotic genome remains transcriptionally inactive. The 8^*th*^ cycle coincides with the migration of nuclei to the periphery of the embryo (forming the syncytial blastoderm). Concomitantly, a first wave of ZGA occurs, leading to the expression of about 60 genes [2], including most of the segmentation genes and the genes required for cellularisation at cycle 14. From then on, the duration of mitotic cycles progressively increases up to 20 minutes at cycle 13. The second wave of ZGA involves over a thousand genes [2,3]. This massive transcriptional activation coincides with a long pause (about 1 h) during the interphase of the 14^*th*^ cycle. During the first thirteen cleavage divisions, the volume of the embryos remains stable while the amount of DNA increases exponentially.

Using haploid mutants (with a nucleo-cytoplasmic (NC) ratio amounting to the half of the wild type one), Edgar et al. [4] have shown that cellularisation was delayed by one mitotic cycle (cycle 15 instead of 14) and proposed that this phenomena was due to the titration of maternal repressors by the increasing amount of DNA. Pritchard et al. [5] highlighted that *fushi-tarazu* repression was dependent on maternal repressor Tramtrack, itself dependent on the NC ratio. More recently Lu et al. [6] have shown that a few zygotic genes are activated depending on the NC ratio. However, a large fraction of the ZGA wave appears to be independent from the NC ratio and rather depends on the maternal clock model, which assumes that the triggering of gene expression depends on the absolute time after fertilisation. The two afore mentioned mechanisms are not exclusive, and they may play complementary roles in ZGA.

Recently, a combination of genetic and functional genomic studies demonstrated a major implication of the factor Zelda between one and three hours after fertilisation [7]. Zelda has been shown to play a role of general transcription amplifier collaborating with Dorsal [8], STAT92E [9], and some other maternal morphogens [10]. This factor binds the TAGteam motif (CAGGTAG), which has been previously proposed to play a role in the activation of pre-cellular blastoderm genes [2,11,12]. The TAGteam motif is overrepresented in peaks obtained from ChIP-seq experiments targeting 21 transcription factors involved in embryonic segmentation [13]. Apart from Zelda, which has been recently shown to be involved in the two waves of ZGA [7], all the other factors reported so far are related with the minor wave. Thus, other factors remain to be identified in order to understand the mechanisms underlying ZGA in Drosophila, including epigenetic regulation.

The goal of our study is to explore the regulatory mechanisms involved in the activation of zygotic genes during the MZT. For this, we started from three transcriptome studies in early Drosophila embryos [2,3,6], selected clusters of genes specifically activated during MZT, discovered over-represented motifs in their regulatory region and predicted cis-regulatory modules comprising combinations of these motifs. Interestingly, this “factor-centric” analysis suggests an important role for Trl, a chromatin-remodelling factor, which led us to further investigate the potential associations between ZGA-associated cis-regulatory modules and various epigenetic marks.

It has been recently established by numerous studies that various types of histone modifications affect transcriptional activation, including methylation and acetylation of histone tails to cite a few [14-17]. Using complementary computational tools, we therefore further investigated the relationship between the presence of binding sites for key transcriptional factors and the presence of different in-vivo histone modifications and DNA binding event, focusing on genomic loci associated with ZGA genes. Our computational results prompt a model that tentatively explains the onset of ZGA by a combination of genetic and epigenetic factors.

## Results and discussion

### Selection of ZGA-responding genes

#### ***Transcriptome studies used in this analysis***

**Figure 2:**
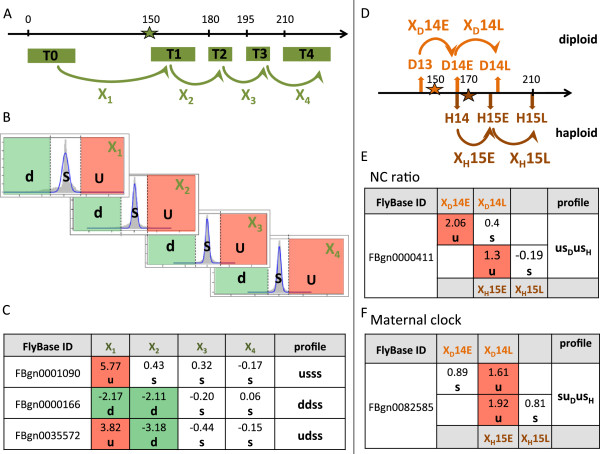
**Discretization of transition profiles(A)** Computation of transition values X1-X4 from successive time points T0-T4 (see Methods for computation) of the dataset from Pilot (2004). The horizontal axis represents time in minutes. The green star indicates the beginning of cellularisation. **(B)** Gene-wise distributions of z-scores (abscissa) computed for each transition. The ordinate represents gene numbers. Dashed lines indicate significance thresholds (*E*-*value* < 0.01), separating down-regulated (d), stable (s) and up-regulated (u) transcripts. **(C)** Correspondence between z-scores profiles and discrete profiles for three representative genes. **(D)**  Computation of transition values applied separately for diploid (D, orange) and haploid (H, brown) genotypes in the dataset from Lu et al. (2009). Stars indicate the beginning of cellularisation. **(E-F)** Combining diploid and haploid transition profiles permits to select genes whose regulation depends on the NC ratio (*us*_*D*_*us*_*H*_, panel E) or on the maternal clock (*su*_*D*_*us*_*H*_, panel **F**.

**Figure 3:**
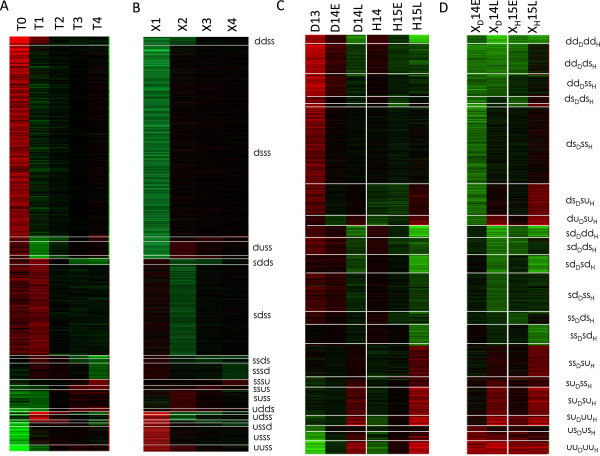
**Clusters from discrete transition profiles.** Heat maps representing transcriptome data from Pilot et al. **(A,B)** and Lu et al. **(C,D)**. Panels **(A,C)** show gene-wise normalized time points (log-ratios of each time point relative to the gene-wise median), whereas **(B,D)** show the corresponding transitions between successive time points. Color scheme: red: positive values, green negative values: black: null values.

In order to identify novel factors involved in ZGA, we have used a series of computational analysis tools to revisit three transcriptomic studies: (1) The first study aimed at detecting genes involved in the process of cellularisation: Pilot *et al.* (2006) [3] extracted mRNAs at five time points corresponding to fertilisation (T0), slow (T1) and fast (T2) phases of cellularisation, early gastrulation (T3) and late gastrulation (T4), respectively (Figure 1B); (2) De Renzis *et al.* (2007) [2] compared the expression profiles of wild-type embryos to those of embryos deleted for half-chromosomes, in order to analyse the respective contributions of maternal and zygotic mRNA during early embryogenesis. They identified five main classes of early expressed genes: (i) maternal and zygotic; (ii) maternal degraded and zygotic; (iii) purely zygotic; (iv) early-activated zygotic; (v) secondary targets (Figure 1C); (3) Lu *et al.* (2009) [6] compared expression profiles in haploid mutants versus wild type embryos in order to distinguish genes regulated by the NC ratio from those controlled by the maternal clock (Figure 1D).

Although these studies addressed distinct questions, the three datasets can be re-analysed and combined to extract genes with marked transcription variations in order to identify specific ZGA regulatory features.

#### ***Discrete transition profiles as signatures of co-expressed gene clusters***

The main computational analysis tools used in this work are encompassed in the flowchart presented in the Additional file 1: Figure S1 and detailed in the Methods section. We first analysed the clusters of co-expressed genes published by Pilot et al. [3] and clusters that we generated ourselves with classical clustering methods (hierarchical and supervised clustering). Published clusters grouped genes with heterogenous temporal profiles (Additional file 2: Figure S2A). After redoing the clustering with optimized parameters, this heterogeneity largely remained (Additional file 3: Figure S3). We therefore decided to apply a custom method (described below and detailed in Methods section) on the temporal profiles from the original studies [3,6]. Transcriptome temporal profiles from [3,6] were converted into “transition values”, defined as the log-ratios between successive time points, which reflect the classical biologist’s perception of changes between developmental stages (Figure 2A). Using a stringent statistical criterion (E-value of a chip-wise normal fit), transition values are converted into three possible discrete classes: up-regulated (u), down-regulated (d) or stable (s), respectively (Figure 2B). Each gene is thereby characterized by a discrete transition profile denoted by a string of the letters u, d and s. Thus, the expression profiles from Pilot [3], which contains five temporal points, were converted into vectors of four transition values and discretized into words of four letters (Figure 2C), which can be easily interpreted as qualitative behaviours. For example, the profile “*usss*” (read “up, stable, stable, stable”) regroups genes whose RNA level increases at the transition between T0 (< 30 minutes after egg laying) and T1 (slow cellularisation phase), and then remains stable: this typically corresponds to zygotically activated genes.

Since these profiles contain four transitions, each with three possible values (u,d,s), a maximum of 3^4^ = 81 distinct strings can be formed. However, only 46 of these 81 profiles are actually represented by at least one gene, among which only 18 are covered by at least ten genes. These 18 profiles and their biological interpretation are listed in Table 1.

**Table 1:** Biological interpretation of the 34 clusters obtained from discrete transition profiles

**Experiment**	**Profiles**	**Nucleo-cytoplasmic ratio NC**	**Gene number**	**Biological interpretation**
Pilot	*ddss*		35	
Lu	*dd*_*D*_*dd*_*H*_	NC	41	Maternal mRNA degraded during cellularisation
Lu	*dd*_*D*_*ds*_*H*_	Maternal clock	165	
Pilot	*dsss*		885	
Lu	*ds*_*D*_*ds*_*H*_	NC	37	Maternal mRNA degraded during slow phase of cellularisation
Lu	*ds*_*D*_*ss*_*H*_	Maternal clock	406	
				
Pilot	*dssu*		11	Maternal mRNA degraded during slow phase of cellularisation
				and zygotic mRNAs transcription during late phase of gastrulation
Lu	*ds*_*D*_*su*_*H*_	Maternal clock	163	
				
Pilot	*dsus*		13	Maternal mRNA degraded during slow phase of cellularisation and zygotic mRNAs transcription during early phase of gastrulation
				
Pilot	*duss*		66	Maternal mRNA degraded during slow phase of cellularisation and zygotic mRNAs transcription during fast phase of cellularisation
Pilot	*sdds*		23	
Lu	*sd*_*D*_*sd*_*H*_	NC	91	
Lu	*sd*_*D*_*dd*_*H*_	Maternal clock	61	Maternal mRNA degraded from fast phase of cellularisation
Lu	*sd*_*D*_*ds*_*H*_	Maternal clock	97	
Pilot	*sdss*		415	
Pilot	*ssdd*		12	
Lu	*ss*_*D*_*sd*_*H*_	Horloge maternelle	111	Maternal mRNA degraded from early phase of gastrulation
Pilot	*ssds*		22	
Pilot	*sssd*		77	Maternal mRNA degraded from late phase of gastrulation
Pilot	*sssu*		28	Zygotic mRNAs transcription during late phase of gastrulation
Pilot	*ssus*		21	Zygotic mRNAs transcription during early phase of gastrulation
Lu	*ss*_*D*_*su*_*H*_	Maternal clock	164	
Pilot	*suss*		75	Zygotic mRNAs transcription during fast phase of cellularisation
Pilot	*suus*		11	
Lu	*su*_*D*_*su*_*H*_	NC	154	
Lu	*su*_*D*_*uu*_*H*_	Maternal clock	47	
Pilot	udss*		23	Transient zygotic mRNAs transcription during cellularisation
Pilot	ussd*		16	
Pilot	usss*		87	
Pilot	uuss*		23	
Lu	*us*_*D*_*us*_H_*	NC	14	Zygotic mRNAs transcription during slow phase of cellularisation
Lu	*us*_*D*_*ss*_H_*	Maternal clock	24	
Lu	*uu*_*D*_*uu*_H_*	NC	60	
Lu	*uu*_*D*_*us*_H_*	Maternal clock	27	

Stars beside the discrete profiles indicate clusters merged into the ZGA cluster.

Regarding the analysis of the data of Lu et al. [6], the transitions between consecutive time points were named by appending the genetic background (denoted by D or H, for diploid or haploid) to the reached time point, with a suffix specifying an early or late stage (E or L respectively). As shown in Figure 2D, transition profiles obtained from Lu experiments in wild type and haploid embryos can be combined in order to distinguish genes responding to the nucleo-cytoplasmic (NC) ratio from those activated by a “maternal clock”. Indeed, genes that depend on NC ratio are expected to respond one mitotic cycle later in haploids than in diploids, since the former embryos contain half the amount of DNA. Thus, the profile “ *us*_*D*_*us*_*H*_” (read “up, then stable diploids, up, then stable haploids”) (Figure 2E) regroups genes activated at transition to the early 14^*th*^ mitotic cycle in diploids (transition *X*_*D*_ 14*E* between time points D13 and D14E), but one cycle later in haploids (transition *X*_*H*_ 15*E* between time points H14 and H15E). In contrast, genes whose activation fit the maternal clock model vary at the same absolute time, irrespective of the DNA amount. For example, genes having the profile *su*_*D*_*us*_*H*_ (Figure 2F) are activated at 165-190 minutes after egg laying in diploids (time point D14L), and at 165-185 minutes in haploid (time point H15E). In total, the 3^2^ = 9 diploid profiles combined with the 3^2^ haploid profiles can form 81 possible transition profiles. However, we obtained only 37 different transition profiles, 24 of which contained at least ten genes. Furthermore, only 16 of them were classified as NC ratio or maternal clock responding genes (Table 1). We left aside the nine remaining clusters because we were not able to interpret the discrete profiles, based on the rules defined in Figure 2E and F.

At this stage, we considered each possible discrete profile as the signature of a distinct gene co-expression cluster (Figure 3). Interestingly, the most populated profiles (18 clusters from Pilot and 16 from Lu containing more than 10 genes) are consistent with ZGA-related behaviours. For example, the clusters Pilot “*dsss*” (885 genes), Lu “ *ds*_*D*_*ss*_*H*_” (406 genes) and Lu “ *ds*_*D*_*ds*_*H*_” (37 genes) correspond to maternal mRNAs degraded during the slow phase of cellularisation, whereas the cluster Pilot “udss” (23 genes) regroups genes showing a transient activation during cellularisation. A list of cluster biological interpretations is provided in Table 1. Strikingly, no gene showed transient activation (“ *ud*_*D*_*ud*_*H*_”) or repression (“ *du*_*D*_*du*_*H*_”) depending on the NC ratio.

#### ***Grouping of co-expression clusters based on discovered motifs***

In addition to the 34 clusters obtained from the discrete transition profiles described in previous section (Additional file 4: Table S1), we included six clusters resulting from the previous published studies: five clusters containing maternal and/or zygotic genes defined by De Renzis and co-workers [2], and one cluster containing genes activated dependently on the NC ratio, defined by Lu and co-workers [6].

In order to detect similarities between clusters containing the same type of genes (i.e. maternal, early or late zygotic genes, etc.) and to regroup the most relevant genes for ZGA regulation analysis, we performed a preliminary discovery of over-represented heptanucleotides [18] in the regulatory regions associated with each of the 40 clusters.

**Figure 4:**
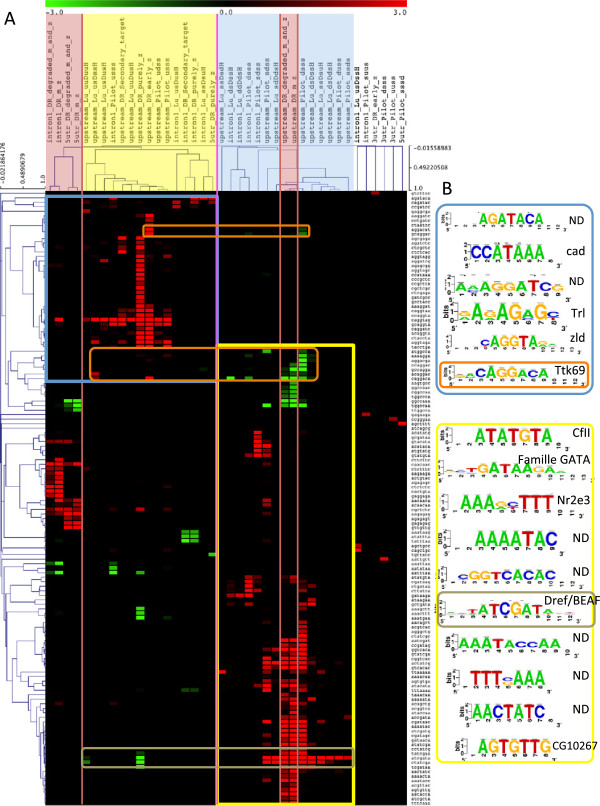
**Motif-based clustering of co-expression clusters (A), and correspondences between discovered and known motifs (B).(A)** Bi-clustering of 7-letters words and gene clusters in function of the under- and over-representation significance of each word in each cluster. Columns correspond to gene clusters, more precisely to the different types of non-coding regions associated to genes contained in clusters (upstream: 5Kb upstream TSS, intron1: first intron, 5utr: 5’UTR, 3utr: 3’UTR). Colors highlight clusters containing genes having the same expression pattern: yellow corresponds to genes significantly activated during the 1st and second wave of ZGA, blue corresponds to genes whose transcripts are maternally provided and significantly degraded during ZGA, red correspond to clusters containing genes whose transcripts are provided maternally as well as zygotically. Clusters with no assigned colors are not classified (unresolved branches) and correspond to non-coding sequences with few significant words. Only clusters containing at least one significant word are shown. Rows correspond to significant 7-letters words in at least one gene cluster. Red and green colors in the heatmap (cells) correspond to over- and under-represented words respectively. **(B)** Motifs resulting from the assembly of clustered overlapping words. Colored squares surrounding groups of motifs correspond to squares in the heatmap that surround words, which were assembled.

Motif discovery was performed separately in upstream non-coding sequences, introns, 5’UTR and 3’UTR in order to cover various types of regulation. The resulting motifs are combined in a matrix containing significance of under- and over-representation of each 7-letters word (represented as lines of the matrix) in each cluster (represented as columns of the matrix). Here, the significance is defined as minus the logarithm of the E-value. We applied hierarchical clustering on the columns of this matrix, in order to regroup co-expression clusters showing similar predicted regulatory motifs. This motif-based clustering revealed three types of clusters (Figure 4A): (i) ’zygotic’ clusters (e.g. Pilot “*usss*”, Lu “ *us*_*D*_*ss*_*H*_”, De Renzis early and purely zygotic, etc.) made of genes activated at early stages of ZGA (the first wave and beginning of the second one; yellow); (ii) “maternal” clusters containing genes whose mRNAs is degraded during early or late cellularisation (blue); (iii) “maternal+zygotic” clusters containing genes transcribed during oogenesis as well as during ZGA (red). This motif-based grouping is consistent with the overlap between clusters in terms of gene composition (Additional file 5: Figure S4A).

The resulting classification tree shows that the clusters containing only zygotically activated genes appear to have a coherent regulation since they cluster tightly, whereas maternal+zygotic clusters reveal a more complex pattern of regulation. Indeed, some motifs over-represented in first introns and 5’UTR of maternal+zygotic clusters are also over-represented in upstream sequences of zygotic clusters (yellow frame on Figure 4A), whereas the motifs discovered in upstream regions of the maternal+zygotic clusters are also found in upstream sequences of maternal clusters (blue frame on Figure 4A). Moreover, clusters containing genes activated during late cellularisation showed none or few motifs and are present at unresolved branches of the hierarchical tree.

We were mostly interested in zygotic activation; the coherent clustering shown by purely zygotic clusters, and the fact that we did not find any specific motif to NC dependent and independent genes led us to merge the ten *uXXX* and zygotic clusters (Pilot “*usss*”, “*udss*”, “*uuss*”,“*ussd*”, Lu “ *us*_*D*_*us*_*H*_”, “ *us*_*D*_*ss*_*H*_”, “ *uu*_*D*_*us*_*H*_”, “ *uu*_*D*_*uu*_*H*_”, De Renzis purely zygotic, early zygotic) into a single cluster totalizing 417 genes, hereafter denoted as “ZGA cluster”, for further analysis (Additional file 5: Figure S4B).

We evaluated the relevance of this newly defined ZGA cluster and analysed the enrichment of these clusters in gene ontology terms (GO biological process, molecular function and cellular component), using the software tool *compare-classes* of RSAT suite [19]. We found 184 significantly enriched terms (E-value <0.01 with a minimal E-value = 8*e*^-31^) in the ZGA cluster that revealed a better enrichment than the purely zygotic (90 terms, minimal E-value = 6*e*^-22^) and early zygotic (6 terms, minimal E-value = 2*e*^-3^). Most of the enriched terms are associated to morphological changes and regulatory processes (Table 2 and Additional file 6: Table S2) that are highly consistent with the developmental embryonic stages studied.

**Table 2:** The 40 most significant associations between the GO terms and genes of the ZGA cluster

**GO identifier**	**GO term definition**	**Nb genes in**	**Genes at**	**GO class coverage**	**e-value**
		**GO class**	**intersection**	**by cluster**	
GO:0009653	BP: anatomical structure morphogenesis	1521	145	10%	8.00E-31
GO:0007275	BP: multicellular organismal development	2739	195	7%	9.50E-30
GO:0048513	BP: organ development	1239	128	10%	4.10E-29
GO:0065007	BP: biological regulation	2287	176	8%	1.20E-28
GO:0048856	BP: anatomical structure development	2734	192	7%	8.40E-28
GO:0032502	BP: developmental process	3056	202	7%	7.60E-27
GO:0009790	BP: embryo development	595	84	14%	1.20E-25
GO:0048731	BP: system development	2161	166	8%	1.60E-25
GO:0050789	BP: regulation of biological process	2075	162	8%	2.70E-25
GO:0045165	BP: cell fate commitment	222	50	23%	8.80E-23
GO:0007389	BP: pattern specification process	512	73	14%	8.90E-22
GO:0048699	BP: generation of neurons	599	79	13%	1.00E-21
GO:0050794	BP: regulation of cellular process	1910	148	8%	3.50E-21
GO:0001071	MF: nucleic acid binding transcription factor activity	301	53	18%	4.80E-21
GO:0003700	MF: sequence-specific DNA binding transcription factor activity	301	53	18%	4.80E-21
GO:0003002	BP: regionalization	479	69	14%	1.40E-20
GO:0048598	BP: embryonic morphogenesis	232	48	21%	5.50E-20
GO:0001709	BP: cell fate determination	123	36	29%	1.10E-19
GO:0006355	BP: regulation of transcription, DNA-dependent	517	68	13%	7.00E-18
GO:0048869	BP: cellular developmental process	1732	133	8%	2.70E-17
GO:0030154	BP: cell differentiation	1695	131	8%	4.00E-17
GO:0051252	BP: regulation of RNA metabolic process	586	71	12%	9.10E-17
GO:0009887	BP: organ morphogenesis	665	76	11%	9.90E-17
GO:0007369	BP: gastrulation	69	26	38%	1.30E-16
GO:0009888	BP: tissue development	532	67	13%	1.70E-16
GO:2000112	BP: regulation of cellular macromolecule biosynthetic process	588	70	12%	5.00E-16
GO:0010556	BP: regulation of macromolecule biosynthetic process	588	70	12%	5.00E-16
GO:0019219	BP: regulation of nucleobase, nucleoside, nucleotide and nucleic acid metabolic process	609	71	12%	8.50E-16
GO:0051171	BP: regulation of nitrogen compound metabolic process	611	71	12%	1.00E-15
GO:0032501	BP: multicellular organismal process	3730	206	6%	1.50E-15
GO:0010468	BP: regulation of gene expression	725	77	11%	4.60E-15
GO:0048729	BP: tissue morphogenesis	305	48	16%	1.10E-14
GO:0031326	BP: regulation of cellular biosynthetic process	633	70	11%	3.20E-14
GO:0048569	BP: post-embryonic organ development	340	50	15%	3.70E-14
GO:0009889	BP: regulation of biosynthetic process	635	70	11%	3.80E-14
GO:0030182	BP: neuron differentiation	514	62	12%	5.60E-14
GO:0060255	BP: regulation of macromolecule metabolic process	838	81	10%	1.40E-13
GO:0060429	BP: epithelium development	291	45	15%	2.90E-13
GO:0009880	BP: embryonic pattern specification	231	40	17%	3.40E-13
GO:0031323	BP: regulation of cellular metabolic process	819	79	10%	4.70E-13

Hypergeometric E-value (expected number of false positives) was computed with the RSAT tool, *compare-classes*[19].

#### ***Zelda, Tramtrack and Trithorax-like binding motifs are over-represented in ZGA genes***

**Figure 5:**
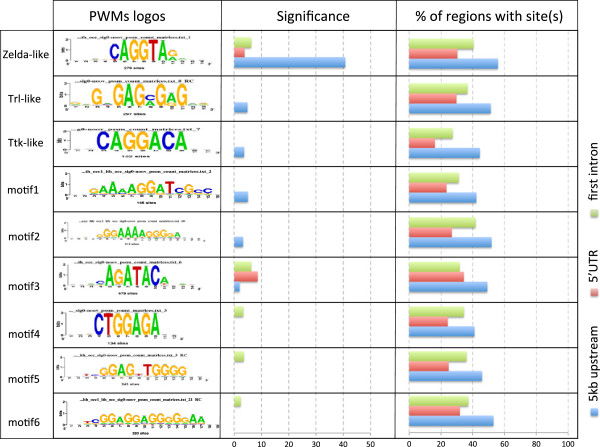
**Selection of the most representative motifs discovered in ZGA non-coding sequences.** Each row represents one discovered motif, represented by its logo. Correspondences with motifs bound by known factors are indicated on the left. For each sequence type (5 kb: 5 Kb upstream TSS, 5’UTR, intron1: first intron), we indicated the significance (“Sig” column), the proportion of region containing at least one occurrence of the motif (“%r / s” column).

In order to understand the mechanisms underlying ZGA regulation, we extended our cis-regulatory motif analysis. The over-represented heptanucleotides found previously were assembled to build position-specific scoring matrices. Figure 4B presents a brief synthesis of the resulting motifs, and their correspondence with known motifs. The most significant motif corresponds to the known Zelda binding motif (significance = 35.54 in purely zygotic genes), detected in upstream regions of the zygotic clusters and in the first introns of the maternal+zygotic cluster. This result is consistent with a recent publication [7], which indicates that Zelda appears to be present in genes activated beyond pre-cellular blastoderm [2,11,12]. In the newly defined ZGA cluster, Zelda motif significance is even higher in upstream regions (significance 40.7), while it is also over-represented in first introns and 5’UTRs (Figure 5). The other motifs are less significant in zygotic clusters but have increased significance in the ZGA cluster. Motif discovery also reported a Trithorax-like (Trl) binding motif in upstream regions of the ZGA cluster, as well as in the first introns and 5’UTR of maternal+zygotic clusters. Trl is a maternal factor acting at different transcriptional levels: it is involved in chromatin remodelling complexes, but also regulates RNA PolII activity by direct interactions with TAF30.

Remarkably, a motif corresponding to the Tramtrack (TTK) binding motif was discovered with the *de novo* approach. TTK is a maternal repressor, which is progressively titrated as the NC ratio increases during early mitotic cycles, thereby releasing the expression of zygotic genes [5]. Surprisingly, the TTK binding motif is found over-represented in the sequences of pre-cellular activated blastoderm genes and of the genes with the discrete signature “Lu *us*_*D*_*ss*_*H*_”, but not in the sequences of genes known to depend on the NC ratio, which might be explained by the intervention of some other factors in this mechanism [5].

The TTK protein has been reported to physically interact with TRL proteins and to repress TRL-mediated even-skipped activation [20]. TTK could act either directly by binding DNA and repressing the transcription of specific target genes, or indirectly by repressing an activator such as Trl. Interestingly, the TTK motif is significantly under-represented (sig = 5) in upstream sequences of maternal+zygotic and maternal clusters. This is consistent with a repressing activity of TTK. Indeed, the presence of TTK binding sites would result in early inactivation in the presence of maternally expressed Ttk. A motif matching the binding motif of Caudal (a maternal factor involved in segmentation) was further detected as over-represented in purely zygotic genes, but not in the ZGA cluster.

Two motifs were discovered in zygotic clusters, as well as in the ZGA cluster, which do not match any annotated transcription factor binding motif (“AGATACA” and “AaAAGGATCG”). However “AGATACA” was previously reported to be involved in chromosome pairing between regulatory regions associated with the mechanism of transvection [21]. It thus seems particularly relevant that the strongest over-representation of this motif was found in 5’UTRs, as well as in upstream sequences. Finally, the analysis of over-represented motifs in the ZGA cluster revealed four more unknown motifs (not discovered in separated zygotic clusters). Logos and significance of all these motifs are displayed in Figure 5. As a control, we performed motif discovery analyses on 410 randomly selected gene clusters (with 41 different sizes) which did not return any of these motifs (cf. Additional file material available on RSAT website at the address (http://rs at.bigre.ulb.ac.be/rsat/data/published_data/Darbo_2013). This confirms the biological relevance of the discovered motifs.

Based on these results, and in order to predict putative cis-regulatory modules (CRMs), we scanned each type of ZGA non-coding sequences with the nine discovered motifs and predicted cis-regulatory modules (CRMs) by detecting cis-regulatory elements enriched regions (CRERs) using *matrix-scan*[22] around ZGA defined genes. We detected 528 CRERs in upstream sequences, 313 in the 5’UTR, and 553 in first introns. Because we retrieved non-coding sequences associated with all alternative transcripts, upstream sequences of the smaller transcripts may overlap first introns or 5’UTR sequences. Moreover, in some genes, the first intron is embedded in 5’UTR. About 70% of the upstream sequences, 50% of the first introns and 40% of the 5’UTR contain at least one CRER (Additional file 7: Table S3). Thus, after having merged the CRERs detected in the different types of regulatory regions, we obtained a final set of 1394 non-overlapping CRERs, hereafter denoted as “predicted CRMs”.

In addition to *de novo* motif discovery, we analysed the enrichment of the ZGA cluster for known motifs, using the program cisTargetX [23]. This tool reveals enriched regulatory features (e.g. motifs or in-vivo datasets) in a set of regions, and ranks these features using a Z-score like enrichements score. Consistently, the results reveal a high enrichment for Zelda (score 12.8) and TRL (score 3.5) binding motifs (Additional file 8: Figure S5). In this analysis, binding motifs for Dorsal (DL), Krüpple (KR) and Bicoid (BCD) were also reported as significantly enriched, which is not unexpected, given the high level of correlation between the binding of these TFs and Zelda. Indeed, a first study of in-vivo binding of BCD, CAD and KR showed that by far the most over-represented motif under the binding peaks was CAGGTAG, hence the binding motif for Zelda [24]. However, many of the CRER do not contain any binding sites for these factors: of the 1394 predicted CRMs, only 765 (54%) contain a predicted binding site for BCD, KR, DL or CAD, using a threshold of *p* = 0.0001 on the binding site. Hence, the observed enrichment for these transcription factors is restricted to a subset of predicted CRMs. This can be confirmed using in-vivo datasets for these factors including Zelda; we restricted our analysis to the CRER containing a predicted Zelda binding site (780 CRER), of which 599 overlap with an in-vivo Zelda binding event, using the ZLD-ChIP datasets published in [7]. Of these Zelda-bound CRER, 423 (70%) do not have any overlap with any of the 21 TFs published in [25]. Hence, Zelda bound in these CRER is not acting as a precursor for segmentation TFs, which are thus likely to be specifically involved in ZGA.

#### ***CRER composition gives insight into ZGA mechanisms***

We analysed the motif composition of the defined CRERs, to get insight into the respective contribution of each motif to the ZGA mechanism. A first observation is that roughly 75% of individual binding sites are contained in CRERs, a percentage that is constant across regions (upstream, intron and 5’ UTR), with the exception of 3’ UTR where this proportion is around 60%. This percentage varies depending on which motif is considered (Additional file 9: Figure S6A). Given that the CRER regions span between 15% and 30% of the regions analysed for motifs, this proportion of motifs in CRER represent a significant enrichment over random expectation, and supports the fact that most of the discovered motif instances are indeed *bona fide* binding sites.

In order to unveil specific organisation patterns, we used a randomization procedure which shuffles the motif instances across CRERs, maintaining the total number of instances of each motif across CRERs, and the number of binding sites in each CRER. A first striking observation is the strong over-representation of homotypic CRER configurations, which is particularly strong in upstream regions (Additional file 9: Figure S6B). As homotypic clusters are known to play an important role in the response to morphogens during early embryogenesis, this is not unexpected, but further supports the validity of the CRERs. The motifs showing the highest over-representations of homotypic clusters are Zelda and the unknown motif AGATACA. For Zelda, the prevalence of homotypic clusters might be a way to respond to “temporal morphogens”, as has been suggested previously [26], while clusters of AGATAC motifs had been identified previously and hypothesized to play a role in chromosome pairing and DNA looping [21]. While heterotypic configurations are globally under-represented, specific combinations are nevertheless found more often than expected, and might point at particular cooperative mechanisms (Additional file 9: Figure S6C). For example, Zelda is found in heterotypic clusters together with either TTK-like motifs or the previously mentioned AGATACA motif, suggesting a mechanism by which distant enhancers bound by Zelda might be brought into contact with promoter regions with the help of mediator proteins binding AGATACA- motifs [21].

### Using in-vivo datasets to investigate epigenetic mechanisms

The motif discovery analysis described in the previous section, and in particular the presence of Trl-related motifs, suggests a possible involvement of epigenetic factors in the activation of zygotic gene expression. In order to complement the previous motif analysis, we thus decided to make use of recently published in-vivo datasets and to investigate epigenetic regulation by analyzing ChIP-seq and DNAse1 accessibility data, using read densities as well as peaks locations. We focused on the factors CBP (0-4 h), Trl (0-8 h) and Zelda (extracted at 1 h, 2 h and 3 h after fertilisation), modified histones (H3K4me1 0-4 h, H3K4me3, H3K9Ac, H3K27Ac, H3K27me3) and open chromatin (DNAse1 accessibility, stage 5).

#### ***Differential motif analysis reveals ZGA specific associations***

After having shown that ZGA CRERs reveal specific associations between motifs, we wanted to investigate whether these associations were general, or ZGA specific. In order to do so, we systematically performed a differential motif analysis with the program *peak-motifs* (RSAT) [27,28] between the ChIP peaks located in non-coding regions associated (hereafter denoted “ZGA-peaks”) and not associated (“non-ZGA peaks”) with genes of the ZGA cluster (Additional file 10: Figure S7 and Additional file 11: Figure S8).

The Zelda binding motif is over-represented in CBP, TRL and DNAse1 ZGA-peaks vs. non-ZGA peaks, confirming the importance of this factor for the control of zygotic genome activation (Additional file 10: Figure S7). The unknown motif AGATACA appears also to be systematically enriched in ZGA vs. non-ZGA datasets, confirming its relevance to ZGA specific processes.

The differential analysis of Zelda-bound regions at different time points shows that the TRL-related motif and AGATACA are highly differentially enriched, underlying the ZGA-specific association between these three motifs. As expected, and as a control of the differential analysis, the Zelda motif does not appear, being present in ZGA as well as non-ZGA peaks.

CBP does not establish direct interactions with DNA, but interacts with a large diversity of DNA-binding transcription factors. In a recent study, the importance of Dorsal for the recruitment of CBP has been shown [29]. Interestingly, in this study, a strong correlation between CBP and TRL binding had also been shown. Here, we do not find Dorsal binding sites over-represented in ZGA vs. non-ZGA CBP peaks. However, a strong enrichment in Zelda binding motif might suggest that Zelda might take over the role of Dorsal for CBP recruitment in the case of ZGA. The TRL motif is found when motif discovery is performed independently on ZGA and non-ZGA CBP peaks (data not shown), showing that CBP and TRL are indeed associated, as noted previously [29]. The fact that the TRL motif does not appear in the differential analysis is likely due to the fact that CBP and TRL co-localize also outside ZGA-specific regions. However, a much stronger overlap between CBP and TRL peaks appears around ZGA-genes: while 18% of TRL-peaks overlapp a CBP-peak between 0-4 h, the proportion reaches 46% when restricting the analysis to peaks located around ZGA-genes.

#### ***High enrichment of CRMs for marks of transcriptional and epigenetic regulation***

The previous analysis indicates the prominent role played by CBP, TRL and Zelda around ZGA-specific genes. We then wanted to investigate in more details the importance of these factors at the precise locations of our predicted CRMs.

In order to detect specific associations, we analysed the densities of reads from ChIP-seq experiments under the 1394 predicted CRMs regions. To evaluate the level of enrichment, we ran the same analysis on a positive control set (114 curated blastoderm-specific CRMs from RedFly) and three types of negative sets: (i) regulatory regions of the 417 ZGA genes scanned with randomized (column-permuted) motifs, (ii) regulatory regions of 417 randomly selected genes, and (iii) 317 CRMs not supposed to be active in blastoderm, according to RedFly annotations.

For each of these datasets, we computed the density of reads under CRMs for various marks of transcriptional and epigenetic regulation: Zelda (global transcription factor), CBP (non-DNA binding cofactor) and TRL (chromatin remodelling factor), histone marks, and DNA accessibility profiles, and compared it with the density of reads under randomly selected regions of similar sizes and types (upstream, intron, …). We also computed a p-value using the Wilcoxon rank test in order to evaluate the difference of enrichment between ZGA CRMs and controls (Additional file 12: Table S4).

**Figure 6:**
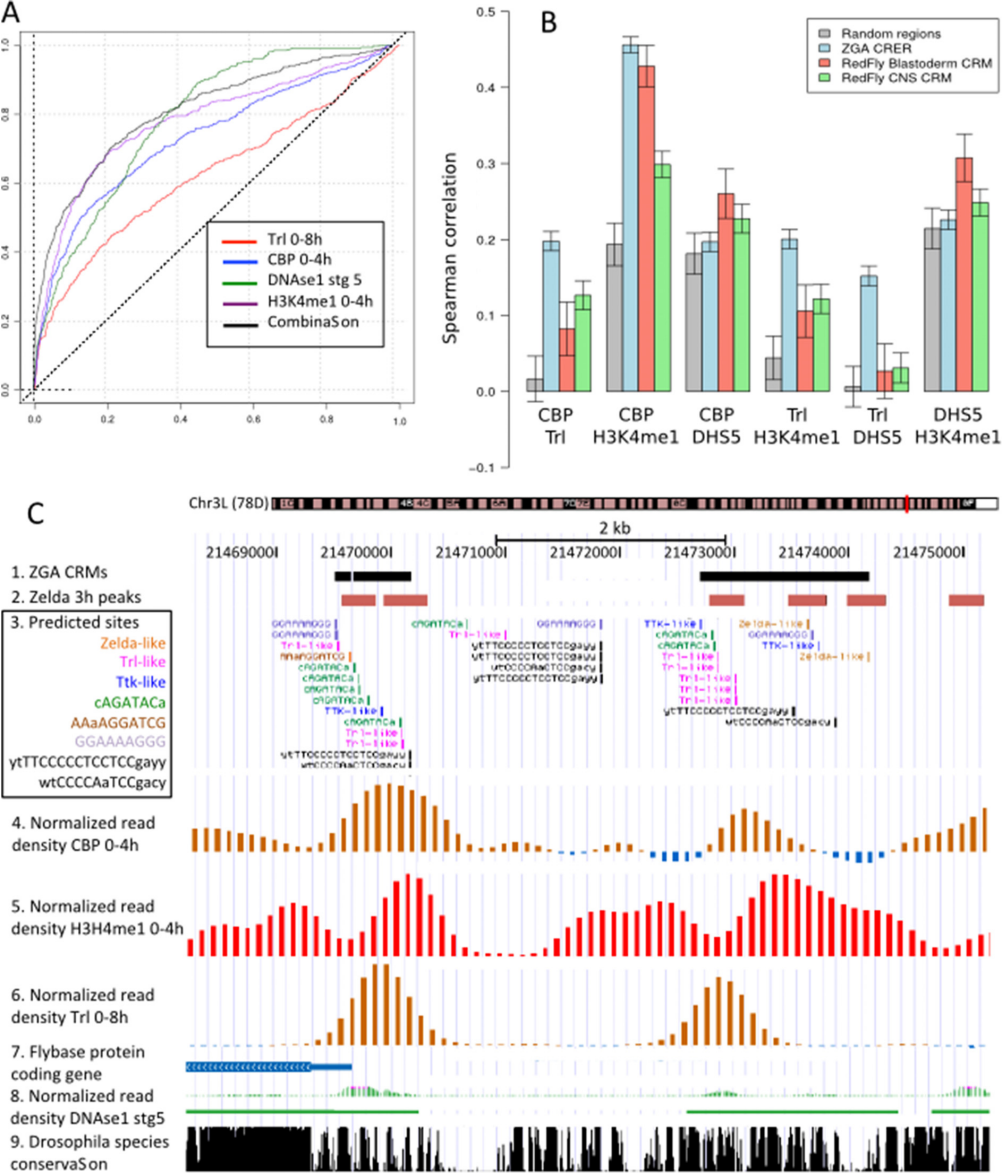
**Combination of four chromatin mark.A.** ROC curves of genome-wise location for ZGA predicted CRMs: Red: Trl 0-8 h; blue: CBP 0-4 h; purple: H3K4Me1; green: DNAse1 accessibility stage 5. The black curve corresponds to the median rank as explained in Methods section. **B.** Pairwise Spearman correlation of the four marks, computed on the regions of the ZGA predicted CRMs (blue), blastoderm CRMs from Redfly (red), central nervous system CRMs from Redfly (green) or on random subsets of regions of identical size and genomic localization (grey). Error bars show the standard deviation over 1000 subsamples. **C.** Example of the 5kb upstream the *crocodile* gene having high density of read for these four marks (1) ZGA CRMs. (2) Zelda 3H peaks. (3) Predicted sites obtained from the scanning of non-coding sequences of ZGA genes with the 9 discovered matrices. (4) Normalized read density produced during CBP ChIP-seq experiment. (5) Normalized read density produced during H3K4me1 ChIP-seq experiment. (6) Normalized read density produced during Trl ChIP-seq experiment. (7) Representation of the 5’ part of *crocodile*. (8) Normalized read density produced during DNAse1 accessibility experiment. (9) Drosophila species conservation.

The results are displayed as ROC curves (Figure 6A), indicating the proportion of CRMs reaching a given density score (ordinate) versus random regions reaching the same score (abscissa). The area under the curve (AUC) was computed to quantify the relative enrichment of different datasets (Additional file 13: Figure S9A). The strongest associations were obtained for CRMs predicted from upstream sequences, as discussed in detail below. However, similar associations were found with CRMs predicted from other sequence types (first introns, 5’UTR and merged CRMs).

The ROC curves (Additional file 14: Figure S10) highlight a strong enrichment of ZGA predicted CRMs for Zelda (1 h, 2 h, 3 h), TRL (0-8 h), CBP (0-4 h) and H3K4me1 (0-4 h) as well as DNAse1 hypersensitive sites (stage 5) that together correspond to signatures of active enhancer. This alone confirms the biological relevance of our CRMs defined purely from sequence motifs around ZGA specific genes. Similar levels of association were found in blastoderm-specific CRMs for marks of active enhancers. However, TRL was found enriched for ZGA CRMs but not for blastoderm-specific CRMs (wilcoxon p-value 8*e*-3). Blastoderm-specific CRMs were also enriched for two repressive marks (H3K9me3 and H3K27me3). This might reflect the tight regulation of the genes controlled by these CRMs, which are active in few spatially located nuclei, but highly repressed by Polycomb-group proteins in the major part of the embryo, as indicated by a recent study by Negre and co-workers [30]. Moreover these repressive marks remain associated with blastoderm CRMs at later stages (Additional file 15: Figure S11).

In contrast, during the time window corresponding to zygotic genome activation (0-4 h), the predicted CRMs of ZGA genes (red curves on Additional file 14: Figure S10) show a significant enrichment for some marks of transcriptional activity (H3K4me1, CBP) but not for repressive marks (H3K27me3, H3K9me3), where the red curve is intermingled with the negative controls (green, purple and blue curves). This seems consistent with a general activation of many genes in the whole embryo.

**Figure 7:**
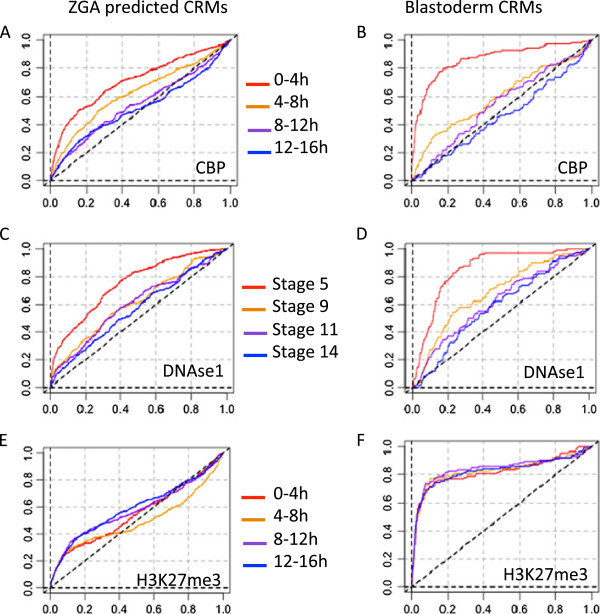
**Dynamics of CRM occupancy by epigenetic marks.** ROC curves representing enrichment of CRMs predicted in ZGA-associated regulatory regions (left panels) and curated blastoderm-specific CRMs (right panels) for CBP **(A,B)**, DNAse1 accessibility **(C,D)** and H3K27me3 **(E-F)**. Red, orange, purple and blue denote different timing, from the earliest to the latest. Time windows for CBP and H3K27me3: 0-4 h, 4-8 h, 8-12 h, 12-16 h; for DNAse 1: developmental stages 5 (∼2 h30), 9 (∼4 h), 11 (∼6 h) and 14 (∼12 h).

Figure 7 shows the ROC curves for CRM occupancy by CBP, DNAse1 and H3K27me3 at successive stages of embryonic development. For both ZGA predicted and blastoderm-specific curated CRMs, CBP occupancy and DNAse1 accessibility are clearly restricted to very early stage (0-4 h) corresponding to the two waves of ZGA (1 h and 3 h, respectively), and rapidly decay at later stages. The same trends are observed for Trl (see AUC distributions for all data sets in Additional file 13: Figure S9B). In contrast, the strong enrichment of repressive mark H3K27me3 in curated blastoderm-specific CRMs is constant during all the studied period (0-16 h). On the downside, comparing the right and left panels reveals that enrichments curves are more pronounced for experimentally validated blastoderm CRMs than for ZGA predicted CRMs, which likely reflects the generation of false positive among the latter.

Previous studies have shown that some of these marks are correlated [31] and do not act independently from each other. Using a computational strategy developed previously [32], we used a ranking approach to compute the correlation between these marks for *(i)* random non-coding regions of the genome matching positional biases of ZGA CRMs, *(ii)* specifically for the ZGA predicted CRMs as well as for *(iii)* blastoderm CRMs from Redfly and *(iv)* central nervous system CRMs from Redfly (Figure 6B; Methods).

Most combinations show a global positive correlation, even in randomly selected regions. Since random regions have been sampled from locations characteristic of ZGA CRER, this reflects a positional effect specific to upstream or intronic regions. The combination CBP/H3K4me1 shows a higher correlation for all three classes of functional elements compared to random regions, as expected from previous studies [30].

However, some combinations show a much higher degree of correlation for ZGA CRERs compared to random regions or other CRMs, notably CBP/Trl and H3K4me1/Trl. The fact that Trl is involved in these ZGA-specific combinations is interesting, as Trl alone is not the best discriminant between ZGA CRERs and other regions (Figure 6A). While Trl and CBP are known to interact [29,30], our results suggest that the synergy between them is even higher on ZGA-specific CRMs and might contribute to the activation of the zygotic genome.

## Conclusion

### From transcriptome data to CRMs prediction and epigenetic context characterisation

The goal of our study was to investigate the mechanism of zygotic genome activation. In order to do so, we *(i)* re-analysed published datasets to carefully define a list of ZGA-related genes, *(ii)* applied motif discovery approaches to uncover potential regulators of this process, and *(iii)* combined in-vivo datasets for various epigenetics factors to understand the interplay between the different regulators of the ZGA.

In particular, using published transcriptome data, we proposed a novel method to cluster gene expression profiles in time-course experiments, which does not require any parameter in order to define co-expression clusters. Functional analysis (expression profiles, non-coding sequence analyses, functional classes enrichment) of the different clusters allowed us to delineate a comprehensive and coherent cluster of genes activated during ZGA. The motifs discovered in the corresponding genes led us to propose several factors and co-factors potentially acting in trans, along with putative cis-regulatory modules.

Analyses of specific associations of predicted CRMs and epigenetic marks led us to propose a model combining different factors (Zelda, TRL, CBP and other unknown factors), which presumably bind accessible and active chromatin regions. In particular, we highlighted to importance of a DNA-motif, AGATACA, which is not yet characterized, but might correspond to a structurally important element or a DNA-binding motif.

From our results, we ranked the predicted CRMs combining TRL, CBP, DNAse1 accessibility, and H3K4me1 data to select the most relevant ones, which can be visualized in their genomic context using the UCSC genome browser. For example, Figure 6C shows the region upstream the TSS of the gene *crocodile*, a purely zygotic gene, whose activation is dependent on the NC ratio and which is involved in the specification of the most anterior head segment.

### Tentative regulatory model and prediction of novel CRMs potentially involved in ZGA control

**Figure 8:**
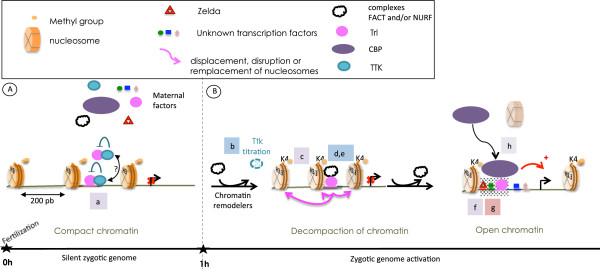
**Integrative model for ZGA combining transcription and epigenetic factors.(A)** Chromatin state and factor organisation before ZGA. **(B)** Chromatin opening and transcriptional activation. Supporting evidences: letters (from a to j) indicate the clues brought by literature (blue), by our bioinformatics treatments (red), or both (purple). (a) See Figure 4, Additional file 15: Figure S11B and [20]; (b) See [5]; (c) See Additional file 15: Figure S11B; (d,e) See [16,17]; (f) See Figure 4, Additional file 10: Figure S7 and [7]; (g) See Figure 4, Additional file 15: Figure S11B; (h) See Additional file 10: Figure S7, Figure 7 and [29].

During the first hour of development, drosophila zygotic genome is transcriptionally silent. As shown in Figure 8A, based on the over-representation of TRL and TTK binding motifs in ZGA non-coding sequences, as well as on TRL binding profile and on previous studies [20], we propose that, before ZGA, TTK could exert a general inhibition on TRL mediated transcription activation through protein-protein interaction.

As TTK becomes titrated by the increasing NC ratio, TRL could be released and become active. Moreover, according to the recent RNA-seq data from Gelbart and Emmert, the amount of Trl mRNA increases from 2h to 4h after egg fertilisation [33]. TTK could thus repress TRL while its abundance is still low, suggesting a mutually enforced effect of TTK titration (NC ratio-dependent) and TRL increase (NC ratio-independent). Binding of TRL could in turn trigger the recruitment of chromatin remodelling complexes. Consistently, we found a high association between predicted CRMs in ZGA-associated regulatory regions, ChIP-seq profiles of TRL binding, and H3K4me1 occupancy.

TRL is not a ZGA-specific factor. What is its exact role during ZGA? While the answer to this question would require experimental validation, our study suggests a mechanism analogous to what has been recently described for dorso-ventral patterning [29], namely that the specificity of TRL action during ZGA might be conveyed by Zelda. This transcription factor has been shown to be primarily involved in the very early stages of embryogenesis, and we find ZGA-specific over-representation of Zelda binding motifs in CBP bound regions around ZGA-genes (Additional file 10: Figure S7). Our model thus involves Zelda, TRL and possibility another factor binding AGATACA. Specific enrichment of the combination of TRL, CBP, H3K4me1 and open chromatin suggests that the global cofactor CBP could be recruited by these factors at the location of the ZGA CRMs. Consistently, both TRL and CBP contain a Q-rich protein-protein interaction domain [34-36], suggesting potential interactions between these two proteins. The high over-representation of TRL binding motif in CBP peaks reinforces this hypothesis. In contrast, the absence of association with acetylated histone 3 suggests that CBP might not act as an acetyl-transferase here, but instead could act as a bridge between the transcription factors and the basal machinery.

## Methods

### High-throughput data

Transcriptome and ChIP-seq data were retrieved from GEO database (http://www.ncbi.nlm.nih.gov/geo/). Transcriptome data: GEO Reference Series IDs GSE3955 [3], GSE14287 [6]. ChIP-seq data: Zelda dataset (GSE30757), other datasets belong to the GSE23537 super series from ModENCODE project [30].

DNAse1 accessibility data were retrieved from Berkeley Drosophila transcription Network Project (http://bdtnp. lbl.gov/Fly-Net/browseAccess.jsp). As the reads from these experiments were mapped on the dm2 assembly, genomic coordinates were converted from dm2 to dm3 assembly with liftOver (http://genome.ucsc. edu/cgi-bin/hgLiftOver).

### Discrete transition profiles

The analysis of microarray data was done with the R statistical package version 2.15.0 [37] and Bioconductor libraries version 1.4.7 [38]. The original datasets extracted from GEO were normalized with the RMA method [39]. RMA-normalized intensities were then converted into discrete transition profiles. We denote as *T*_*i*,*j*_ the median value of the 3 replicates for gene i at time point *j*. The transition value *X*_*i*,*j*_ of gene *i* from time point *j*-1 to time point *j* is computed as follows (Figure 2A).

(1) $$X_{i,j}=\text{lo}g_{2}(\frac{T_{i,j}}{T_{i,j-1}})$$

Transition values are converted to Z-scores using robust estimators to avoid the effects of outliers.

(2) $$Z_{i,j}=\frac{X_{i,j}-{\tilde{m}}_{j}}{{ŝ}_{j}}=\frac{X_{i,j}-{\tilde{m}}_{j}}{{\left(\text{IQR}/1.349\right)}_{j}}$$

where *m~* is the median, ${ŝ}_{j}$ is the estimated standard deviation that corresponds to the observed inter-quantile range (IQR) of the transition values to time point *j*, standardized by the IQR of a standard normal distribution (*mean* = 0, *s* = 1) which equals 1.349. The P-values of Z-scores are computed according to the standard normal distribution, and converted to E-values.

(3) $$\text{Eva}l_{i,j}=\text{Pva}l_{i,j}*G$$

where *G* is the total number of genes. Z-scores are discretized by applying a stringent threshold Θ_0.01_ corresponding to an E-value of 0.01 (Figure 2B).

(4) $$\begin{array}{llll} & & D_{i,j}=u\;\text{if}\;Z_{i,j}\geq \Theta_{0.01} & \\ & & D_{i,j}=d\;\text{if}\;Z_{i,j}\geq \Theta_{-0.01} & \\ & & D_{i,j}=s\;\text{otherwise} & \end{array}$$

Each gene is thus characterized by a discrete transition profile described as a vector of letters u,d,s (Figure 2C).

### Functional enrichment

Functional enrichment analyses were performed with compare-classes (RSAT, http://rsat.ulb.ac.be/rsat/) [19].

Gene ontology (Revision 1.2125) and gene-GO associations (version fb_2011_08) were retrieved from Flybase (http://flybase.org/static_pages/downloads/bulk data7.html) [40]. We discarded the association with low evidence code: NAS (Non-traceable Author Statement), NR (Non-Recorded) and ND (No biological Data available). Each ontology class (molecular function, biological process, and cellular component) was analysed separately. The significance of the enrichment is estimated with the hypergeometric p-value, corrected for multi-testing by computing an analysis-wise E-value:

(5) Eval=Pval∗n

where *n* is the total number of comparisons between a GO class and a gene cluster. To avoid under-estimating the significance, only genes with at least one annotation in GO were considered for this analysis (the “population size” parameter of compare-classes was set to the number of *Drosophila melanogaster* genes annotated in GO, while the non-annotated genes were discarded from the clusters for this enrichment analysis step).

### Analysis of regulatory sequences

The analysis of regulatory sequences relied on the Regulatory Sequence Analysis Tools (RSAT, http:// rsat.ulb.ac.be/rsat/) [27,41] and CisTargetX (http://med. kuleuven.be/cme-mg/lng/cisTargetX/) [23].

#### ***Sequence retrieval***

We used the tool retrieve-ensembl-seq [42] to retrieve non-coding sequences associated to each *Drosophila melanogaster* gene (upstream, 5’UTR, 3’UTR, first intron). Upstream non-coding sequences were extracted up to the closest neighbor gene, with a maximal length of 5 kb. We activated the options to mask coding sequences and repeats, as well as options to retrieve non-coding sequences for all alternative transcripts and to merge overlapping ones.

#### ***Motif discovery***

To automatize motif discovery on the various non-coding sequence types for the different clusters defined during this study, we used the script gene-cluster-motifs, a task manager available in the stand-alone version of RSAT. Among the different motif discovery algorithms supported by this task manager, we ran oligo-analysis [18] and dyad-analysis [43].

These algorithms are based on words and dyads counting respectively. The number of occurrences of each word (dyad) is compared to the expected frequencies observed in a reference sequence set. Specific background models were built for each sequence type (upstream, first intron, 5’UTR, 3’UTR) by computing oligonucleotide and dyad frequencies in the whole set of genomics sequences of the same type. Significance of over-representation is estimated using binomial distribution by computing a nominal p-value.

Over-represented words (oligos) and spaced word pairs (dyads) were assembled and converted to position-specific scoring matrices with the tool matrix-from-patterns (RSAT).

An important advantage of word-based approaches is their scalability: the computing time increases linearly with sequence size, in contast with machine-learning approaches such as MEME or Gibbs motif sampler, whose complexity is quadratic or worse (see [27] for a quantitative evaluation of time efficiency).

Finally, discovered motifs were compared to motif databases (JASPAR: http://jaspar.genereg.net/[44], FlyFactorSurvey: http://pgfe.umassmed.edu/TFDBS/[45]) with compare-matrices (RSAT).

#### ***Peak-motifs***

Peaks from genome-wise location studies were analysed with peak-motifs [27,28] (RSATools).

We ran all motif discovery algorithms available in the web site (*oligo-*, *position-*, *local-word-* and *dyadanalysis*). We searched for over-represented 6- and 7-mers(*oligo-,position-*, *local-word-analysis*) and for pairs or trinucleotides spaced by 0 to 20 nucleotides (*dyad-analysis*). Background was computed from input sequences using a markov model of *k* - 2 with k representing the oligomer length (*oligo-*, *dyad-analysis*). We selected JASPAR Core Insects, DMMPMM and iDMMPMM motif databases for comparison of discovered motifs with known binding motifs.

#### ***Motif enrichment***

CisTargetX was used with default parameters, excepting the parameter “Z-score threshold”, for which we selected the option “Determine threshold automatically” instead of the 2.5 default value.

#### ***Cis-Regulatory element Enriched Regions (CRERs)***

CRERs were predicted with matrix-scan (RSAT) [22]. To compute CRERs significance, we kept sites with a maximal p-value of 10^-4^, and imposed a distance of at least six nucleotides between consecutive sites to discard overlapping sites that would bias the computed significance. The CRERs length was allowed to vary from 30 and 800 bp. Only CRERs with at least a significance of 2 were further analysed. The background models were computed from input sequences using a Markov model of order 2.

### Enrichment of CRMs in ChIP-seq reads

To compare predicted CRM and ChIP-seq profiles, we defined a method to integrate the density of reads over a given region. As a negative control, we measured the read density under random selections of genomic regions of the same sizes as the CRMs. The distributions of densities were compared with ROC (receiver-operating characteristic) curves. The random regions were generated from upstream, first intron, and 5’UTR locations in accordance to the analysed set of predicted CRMs.

#### ***Computation of the intensity under a region (I***_***r***_)

We used the WIG files available in GEO, which contains the ChIP density values at regularly spaced positions (one value every 10 or 100bp depending on the ChIP-seq experiment). To measure the enrichment of a given region of interest (e.g. predicted CRM) for a given ChIP-seq annotation track, we interpolated densities between the annotated positions, and sum their values over the whole length of the region, to obtain a total read intensity of the region (*I*_*r*_). Additional file 16: Figure S12A presents the principle and notations used in following formulas. Let us consider a pair of consecutive annotated positions *x*_*i*_ and *x*_*i*+1_ (separated by 100bp for example) with densities *d*_*i*_ and *d*_*i*+1_, respectively. Under linear interpolation, the sum of densities of all the nucleotide positions between them equals the area of a trapezoid delimited by the density values at *x*_*i*_ and *x*_*i*+1_. The integrated intensity (*H*_*i*,*i*+1_) between these two successive reads is thus computed as follows.

(6) $$H_{i,i+1}=\left(\right)\frac{d_{i}+d_{i+1}}{2}))(x_{i+1}-x_{i})$$

Since the start and end of the region of interest does not always coincide with the precise positions of spaced reads, we interpolate the density at the start position of the region (*d*_*s*_).

(7) $$d_{s}=d_{0}+\frac{x_{s}-x_{0}}{\left(x_{1}-x_{0})(d_{1}-d_{0}\right)}$$

where *x*_*s*_ is the starting position of the region of interest.

In the same way, we estimate the read density (*d*_*e*_) at the end position (*x*_*e*_) of the region:

(8) $$d_{e}=d_{n+1}+\frac{x_{e}-x_{n}}{\left(x_{n+1}-x_{n})(d_{n+1}-d_{n}\right)}$$

where *x*_*n*_ and *x*_*n*+1_ are the discrete read positions just before and after *x*_*e*_, respectively, and *d*_*n*_ and *d*_*n*+1_ the corresponding read densities.

We then compute integrated densities *H*_*s*_ between the region start (*x*_*s*_) and the first annotated read under the region (*x*_1_)

(9) $$H_{s,1}=\left(\right)\frac{d_{1}+d_{s}}{2}))(x_{1}-x_{s})$$

as well as the integrated density *H*_*e*_ between the rightmost annotated read under the region (*x*_*n*_) and the region end (*x*_*e*_)

(10) $$H_{n,e}=\left(\right)\frac{d_{n}+d_{e}}{2}))(x_{e}-x_{n})$$

We can thus compute the integrated read density under the whole region (*H*_*R*_):

(11) $$H_{R}=H_{s}+\overset{n-1}{\underset{i=1}{\sum }}H_{i,i+1}+H_{e}$$

The average region density (*D*_*R*_) is obtained y dividing this integrated density by the region length (*L*_*R*_):

(12) $$D_{R}=\frac{H_{R}}{L_{R}}$$

#### ***Generation of random regions***

For each CRM type (predicted or curated), we generated ten replicates of random regions of the same lengths as the original CRMs. For each sequence type (upstream, first intron, 5’UTR, 3’UTR), the random regions were retrieved from the whole set of sequences of the same type found in the Drosophila genome. For curated CRMs, random regions were retrieved from upstream sequences since they are almost all present in upstream sequences.

#### ***ROC curves***

The computation of ROC curves is based on region ranking according to *I*_*r*_ as shown in Additional file 16: Figure S12B. Values were then normalized along the x and y axis in order to obtain comparable ROC curves between different analyses, i.e. different tested regions (predicted CRMs from ZGA or control non-coding sequences, curated CRMs etc) or different genome-wide protein location experiments. Area under curves (AUC) were computed for the 1000 first ranks. Ranking of regions based on a combination c of a set *ω* of different genome-wide protein location experiments were computed as follows.

(13) $$k_{c}={\tilde{m}}_{k_{\omega }}$$

where *k*_*c*_ is the resulting rank of a given region of a combination c of the experiments in set *ω* and ${\tilde{m}}_{k_{\omega }}$ is the median of the ranks assigned to the region for all experiments in *ω*.

### Correlation between marks

Following a previous publication [32], we used a complete partition of the Drosophila non-coding genome representing about 136 K regions, and scored these regions with the marks of interest (CBP, Trl, H3K4me1 and DNAse1 HS sites). All 136 K regions were ranked according to these four features. Next, we extracted the subset of regions overlapping the ZGA CRERs, and computed the Spearman correlation between the ranks of these regions for all pairs of features over 1000 subsamples of 80% of the regions. For sake of comparison, we have extracted the CRMs annotated with the terms *Blastoderm* (226 regions) or *Central nervous system* (397 regions) from the Redfly database and performed the same analysis. The barplots on Figure 6B show the mean correlations over the 1000 subsamplings and the error bars indicate the standard deviations. As a negative control, one thousand random regions were sampled from the set of 136 K regions, such that the proportion of upstream and intronic regions matches those of the ZGA CRERs. For each pair of features, the mean and standard deviation of the correlation were computed and plotted.

### Additional file information

All data used in this study and the results are available on the supporting Web site (http://rsat.bigre.ulb. ac.be/rsat/data/published_data/Darbo_2013).

## Abbreviations

AUC: Area under curve; ChIP-seq: Chomatin Immuno-Precipitation and sequencing; CRER: Cis-regulatory element enriched region; CRM: Cis-regulatory modules; GO: Gene ontology; miRNA: micro RNA; mRNA: messenger RNA; MZT: Maternal-to-zygotic transition; NC: Nucleo-cytoplasmic; TF: Transcription factor; TSS: Transcription start site; TFBS: Transcription factor binding site; UTR: Untranslated region.

## Competing interest

The authors declare that they have no competing interests.

## Authors’ contributions

ED contributed to the definition of the analytic workflow, performed the bioinformatics analysis, interpreted the results, generated the tables and figures, and wrote the first version of the manuscript. The manuscript was revised and modified by all authors. The scientific project was initially conceived by DT and TL, and subsequently supervised by JvH and DT for the statistics and bioinformatics and TL for the biological interpretation of the results. The R scripts for statistical analysis of microarray data were developed by JvH and ED. JvH adapted some preexisting tools of the RSAT suite for this specific analysis. CH and ED performed the analysis of enrichment with CisTargetX and epigenetic marks. All authors read and approved the final manuscript.

## Authors’ information

This research was the central part of PhD thesis of ED, under the co-direction of DT and JvH in the TAGC laboratory. CH is Maître de Conférences at Aix-Marseille Université. His research activities consist in conceiving, developing, evaluating and applying bioinformatics approaches to analyse regulatory sequences. TL is a group leader in the institute of developmental biology of Luminy Marseille (France). His research activities consist in the understanding of the cell biological basis of cellular organisation and polarity using a combination of genetic, genomic, bio-physical and cell biological techniques. DT was Professor of Bioinformatics at the Université de la Méditerranée (Marseille, France) until January 2010, and is currently Professor of Systems Biology and group leader at the Institute of Biology of the Ecole Normale Supérieure (Paris, France). JvH was Professor at the Université Libre de Bruxelles (Brussels, Belgium) until Oct 2011, and is now Professor at Aix-Marseille Université (Marseille, France). His research activities consist in conceiving, developing, evaluating and applying bioinformatics approaches to analyse regulatory sequences and biomolecular interaction networks.

## Supplementary Material

Additional file 1: Figure S1 Computational analysis flow chart. Expression and chromatin modification data were retrieved from public databases, and relevant pre-computed datasets were collected from the literature (light yellow boxes). Modules (light red boxes) contain processes (bold case), tools (red boxes) and output results (dark yellow boxes). Asterisks denote custom treatments specifically developed for this analysis. A grey square embeds the steps that have been processed twice (1st step with all clusters: blue arrow, 2nd step with ZGA cluster: green arrow). CRMs: Cis-Regulatory Modules; TSS: Transcription Start Site. Click here for file

Additional file 2: Figure S2 Expression profile visualization of published clusters [3] and clusters obtained from discrete transition profiles. A-B: Left panels: heatmaps representing expression profiles from T0 to T4. Red, green and black indicate expression over, under or equal to the median value along the five time points. Middle left panel: temporal profiles. x-axis indicates the time points, y-axis indicates the log2 signal value, the green line corresponds to the mean signal value, the dashed purple line corresponds to the standard deviation, each grey line represents a gene in the cluster. Middle right panels: transition profiles. x-axis indicates the transitions X1 to X4 between consecutive time points, y-axis indicates the log ratio signal value, each blue circle represents a gene. A. Right panels: Schematization of expression profiles of all clusters defined by Pilot et al., the numbers over the curves indicate the number of genes.The colors of the curves correspond to the vertical line colors in the other panels. B. Right panel: heatmap representing the transition profiles from X1 to X4. Red, green and black indicate expression up-, down-regulation or stability of expression during the variations. Click here for file

Additional file 3: Figure S3 Expression profile visualization of clusters obtained from Pilot at al. [3] data using classical clustering methods. Heatmap representing expression profiles from T0 to T4. Red, green and black indicate expression over, under or equal to the median value along the five time points. A. Hierarchical clustering using dot product metrics and complete linkage. B. One of the cluster obtained with K-means partitioning (*a priori* 10 clusters) by 50 iteration. C. Hierarchical clustering using euclidian distance and complete linkage. Middle panel: temporal profiles. x-axis indicates the time points, y-axis indicates the log2 signal value, the green line corresponds to the mean signal value, the dashed purple line corresponds to the standard deviation, each grey line represents a gene in the cluster. Right panel: transition profiles. x-axis indicates the transitions X1 to X4 between consecutive time points, y-axis indicates the log ratio signal value, each blue circle represents a gene. Click here for file

Additional file 4: Table S1 Summary of clusters composition. Fist sheet (Cross-table): Row names indicate FlyBase IDs of 3411 genes present in at least 1 cluster and columns headers indicate 40 published and discrete profile clustering method obtained clusters. The first word indicates the first author of the study from which data were retrieved. [2,3,6]. Following words indicate cluster names. The last column indicates the number of clusters in which the genes is found. The second sheet (Gene - Cluster) contains the same information as the first one but presented as an association table where each row associates a gene with a cluster. Click here for file

Additional file 5: Figure S4 (A) Gene content comparison between clusters of co-expressed genes in function of their significant overlap. Colors highlight clusters containing genes having the same expression pattern: yellow denotes genes significantly activated during the 1st and early second wave of ZGA; orange denotes genes lately activated (end of cellularisation and gastrulation); blue and green denote genes whose transcripts are maternally provided and significantly early and lately degraded, respectively; finally, red denotes clusters containing genes whose transcripts are provided both maternally and zygotically. Uncolored clusters were extracted from the data of Lu et al. and do not correspond to any known regulatory mechanisms (maternal clock, NC ratio). Lines in the heatmap highlight the significant overlapping between gene clusters. Color scale is represented by a diagonal black to purple gradient corresponding to significance from 0 to 3 (and beyond). (B) Venn diagram representing the overlapping between De Renzis et al. [2] early and purely zygotic gene published clusters (green) and the merged set of genes activated during ZGA derived from the discretization analysis (“uxxx” genes in red). This grouping forms the “ZGA cluster” containing 417 genes. Click here for file

Additional file 6: Table S2 Summary of enriched GO terms in clusters. The first sheet lists the enrichment results obtained for ZGA cluster. The second sheet corresponds to enrichment results obtained for all analysed clusters. The third sheet contains the results for ZGA genes carrying at least one CRER. Each table summarizes the number of tested genes in each cluster for given GO classes (MF: molecular function, BP: biological process, CC: cellular component) and the total number of genes contained in the corresponding class; e-value corresponds to the p-value corrected for multi-testing (see Methods), while significance is a log2 transformation of the e-value. Click here for file

Additional file 7: Table S3 Summary of information relative to matrix-scan predicted CRERs. The first sheet summarizes the statistics of the occurrences of CRERs (predicted CRMs) in non-coding regions of ZGA genes. Each row corresponds to a CRER. The names of following sheets indicate the type of the non-coding sequences analysed. The last sheet (“CRERs coordinates”) contains coordinates of merged CRERs. Click here for file

Additional file 8: Figure S5 (A) Summary of CisTargetX results. The blue curves of the ROC graphs represent the ranking of ZGA genes (ordinate) among all *Drosophila melanogaster* genes (abscissa) (see [23] for details). The red curve represents the mean of the scores for all matrices of the reference databases, and the green curve indicates a confidence interval (2 sd from the mean curve). The colors of the lines match that of the contours of the corresponding binding motifs (the use of several motif databases generates redundancy). The logo displayed corresponds to the motif with the best enrichment score within the group of similar motifs. Under each logo, the corresponding transcription factor is specified. Click here for file

Additional file 9: Figure S6 Organization of CRERs. (A) Between 60% and 80% of the motif instances lie in CRERs, which represents a significant enrichment over random expectation, given that the CRER span only 15 to 30% of the regions considered (dashed lines). (B) Homotypic CRERs are found significantly more often than expected from a randomization procedure preserving overall motif frequency and CRER motif density. This enrichment is particularly pronounced in the upstream regions. Shown is the p-value based on a Poisson distribution of expected number of instances. (C) Significance of homo-/heterotypic configurations. The first three patterns correspond to the known Zelda, TRL and TTK motifs. Zelda (CAGGTA) and AGATACA motif show striking enrichment in homotypic configurations, while heterotypic configurations containing Zelda together with either TTK (CAGGACA) or AGATACA-motif are significantly more frequent than expected. Numbers indicate the significance, i.e. -*log*10(*Q*-*value*). Click here for file

Additional file 10: Figure S7 *peak-motifs* differential analyses between ZGA and non-ZGA peaks for CBP, TRL, H3K4me1 and DNAse1 accessibility. The circle inclusions indicates the subset of peaks overlapping coding sequences of the ZGA genes (dark blue circle) relative to the total peak set (green circle). Circle surfaces are proportional to the numbers of peaks (indicated besides the circles using the same color code). This representation makes clear that the large majority of peaks fall into non coding regions. For each experiment, we indicate the number of Drosophila genes and ZGA genes containing at least one peak, and the binomial p-value of the enrichment of peaks in ZGA non-coding regions according to the expected frequency of peaks per nucleotide (all Drosophila non-coding sequences). The last column summarizes the results (logo of over-represented motifs, their significance and the percentage of peaks carrying at least on motif occurrence) of the differential analysis performed with *peak-motifs* between ZGA versus non ZGA peaks. Click here for file

Additional file 11: Figure S8 *peak-motifs* differential analyses between ZGA and non-ZGA peaks for Zelda. Confer to Additional file 10: Figure S7 legend. Click here for file

Additional file 12: Table S4 Results of the Wilcoxon rank-sum test computed for the 38 ChIP-seq/DNAse1 experiments and the five types of CRMs. Each row corresponds to an experiment and each column to a type of CRMs (ZGA, Redfly blastoderm, Redfly non blastoderm, permuted matrices, random genes). Click here for file

Additional file 13: Figure S9 AUC measuring the capability of various epigenetic marks to discriminate ZGA regions and CRM from random selections. Distribution of AUC values (ordinate) obtained from 38 genome-wise location experiments (abscissa) and predicted CRMs from different type of ZGA non-coding sequences (A) or predicted CRMs in ZGA upstream sequences, blastoderm CRMs from RedFly and negative controls (B). Click here for file

Additional file 14: Figure S10 ROC curves showing the enrichment in reads for various types of genomic regions (predicted CRMs, annotated CRMs, random controls). The ordinate and abscissa represent respectively the fractions of test regions (Sensitivity) and random regions (False Positive Rate) passing a given threshold of density. The kind and time window of each dataset is specified in the right corner. Different line colors denote different types of test regions. Black: 114 CRMs annotated in RedFly database as enhancing expression in the blastoderm embryo; purple: 317 CRMs supposed to be silent in early embryo, according to RedFly annotations; red: 528 CRMs predicted by scanning the 5kb upstream regions of the ZGA genes with nine discovered motifs; blue: 164 CRERs predicted by scanning the 5kb upstream regions of 417 random genes with the same matrices; green: 151 CRERs predicted by scanning the 5kb upstream regions of the ZGA genes with nine randomly column-permuted matrices. Click here for file

Additional file 15: Figure S11 ROC curves representing enrichment of CRMs for repressive marks and evolution along the development. A-C: Black: 114 CRMs annotated in RedFly database as enhancing expression in the blastoderm embryo; purple: 317 CRMs supposed to be silent in early embryo, according to RedFly annotations; red: 528 CRMs predicted by scanning the 5kb upstream regions of the ZGA genes with nine discovered motifs; blue: 164 CRERs predicted by scanning the 5kb upstream regions of 417 random genes with the same matrices; green: 151 CRERs predicted by scanning the 5kb upstream regions of the ZGA genes with nine randomly column-permuted matrices. D-F: Red, orange, purple and blue denote different timing from the earliest to the latest. Click here for file

Additional file 16: Figure S12 Principle of the analysis of region enrichment in reads. (A) Illustration of values used for the computation of the intensity under a given region *I*_*r*_ as defined in Methods section. The histogram represents the read density tracks. Green read density tracks represent negative values (the lowest is indicated in the margin in green), while red read density tracks represent positives ones (the highest is indicated in the margin in red). The black curve represents the linear extrapolation of read density under the region. (B) The table lists the genomic positions (chromosome, start, end), *I*_*r*_, the type (random or regions to test) and the corresponding rank for each region considered. The ROC curve (right) displays the cumulative numbers of random (abscissa) and test (ordinate) regions found according to their ranking. Click here for file
