# Supplementary material for: Transcriptional and epigenetic signatures of zygotic genome activation during early drosophila embryogenesis
Source: BMC Genomics. 2013 Apr 5;14:226. doi: 10.1186/1471-2164-14-226 (PMC3706223; doi:10.1186/1471-2164-14-226)
Supplement: Additional file 1: Figure S1 — Computational analysis flow chart. Expression and chromatin modification data were retrieved from public databases, and relevant pre-computed datasets were collected from the literature (light yellow boxes). Modules (light red boxes) contain processes (bold case), tools (red boxes) and output results (dark yellow boxes). Asterisks denote custom treatments specifically developed for this analysis. A grey square embeds the steps that have been processed twice (1st step with all clusters: blue arrow, 2nd step with ZGA cluster: green arrow). CRMs: Cis-Regulatory Modules; TSS: Transcription Start Site. [file 1471-2164-14-226-S1.pdf]

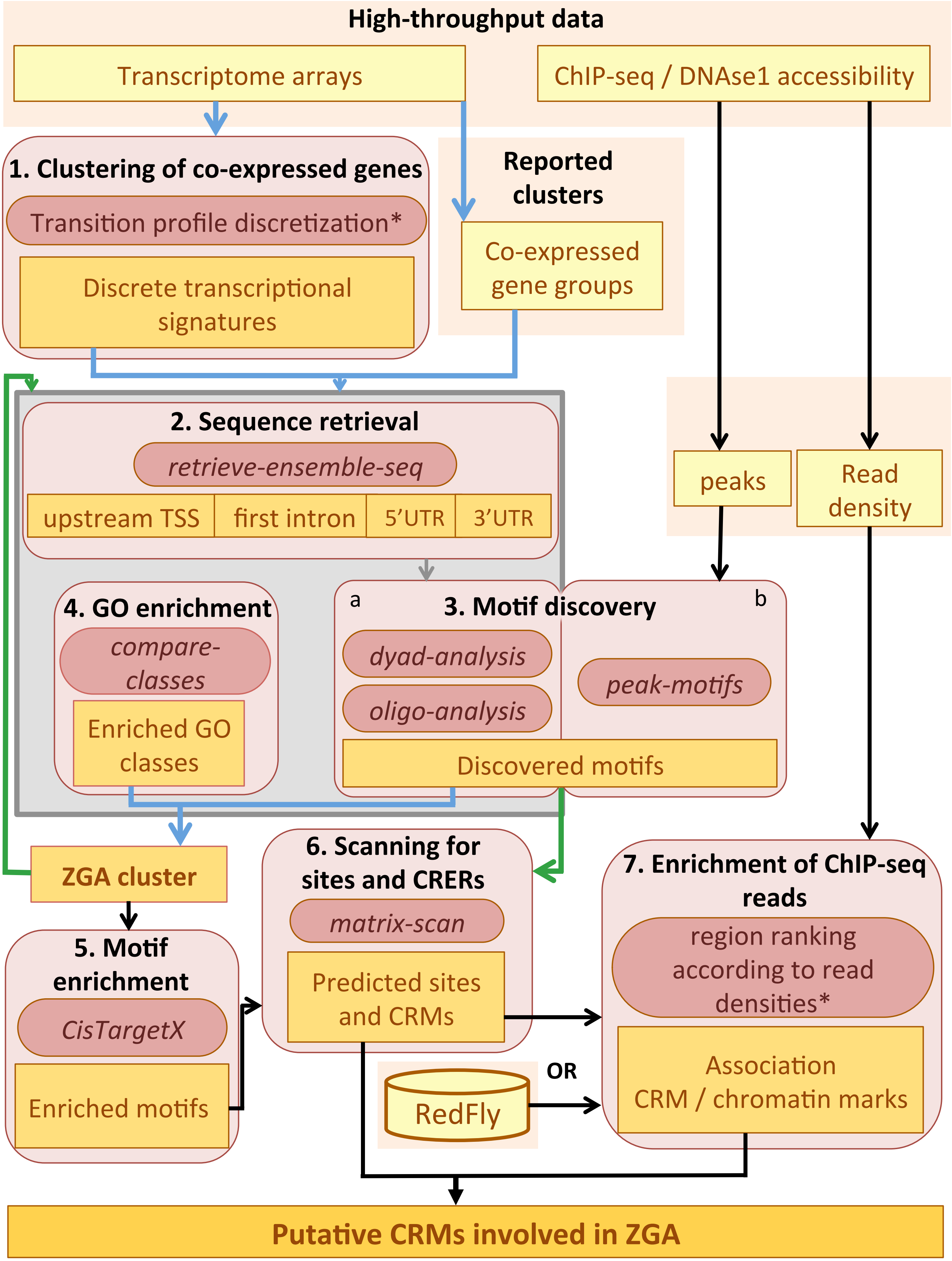
High-throughput data
Transcriptome arrays
ChIP-seq / DNAse1 accessibility
1. Clustering of co-expressed genes
Transition profile discretization*
Discrete transcriptional signatures
Reported clusters
Co-expressed gene groups
peaks
Read density
2. Sequence retrieval
retrieve-ensemble-seq
upstream TSS
first intron
5'UTR
3'UTR
4. GO enrichment
compare-classes
Enriched GO classes
a
3. Motif discovery
b
dyad-analysis
oligo-analysis
peak-motifs
Discovered motifs
ZGA cluster
6. Scanning for sites and CRERs
matrix-scan
Predicted sites and CRMs
7. Enrichment of ChIP-seq reads
region ranking according to read densities*
Association CRM / chromatin marks
5. Motif enrichment
CisTargetX
Enriched motifs
OR
RedFly
Putative CRMs involved in ZGA
