# Supplementary figures and images for: Transcriptional and epigenetic signatures of zygotic genome activation during early drosophila embryogenesis
Source: BMC Genomics. 2013 Apr 5;14:226. doi: 10.1186/1471-2164-14-226 (PMC3706223; doi:10.1186/1471-2164-14-226)

A

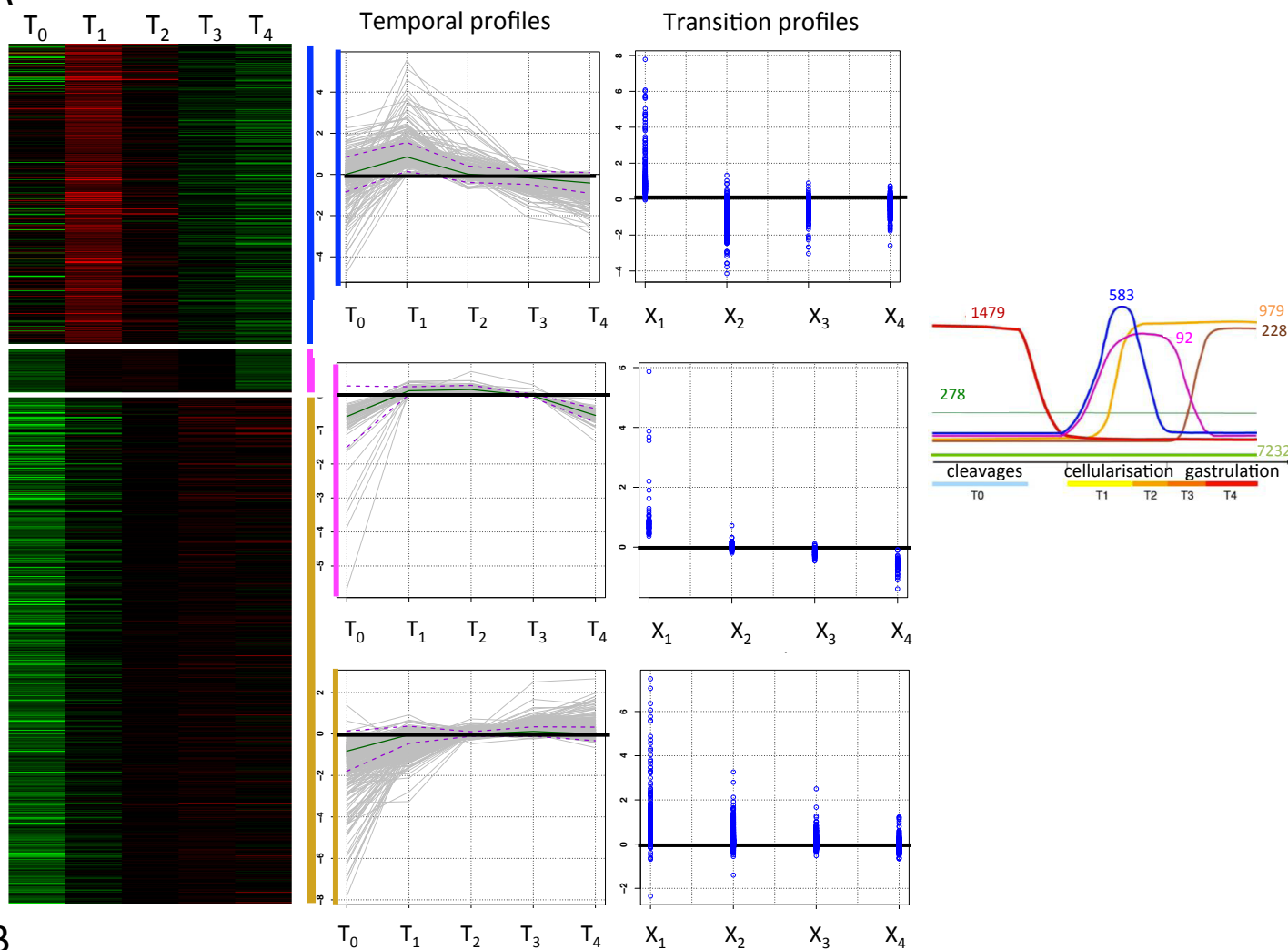

B

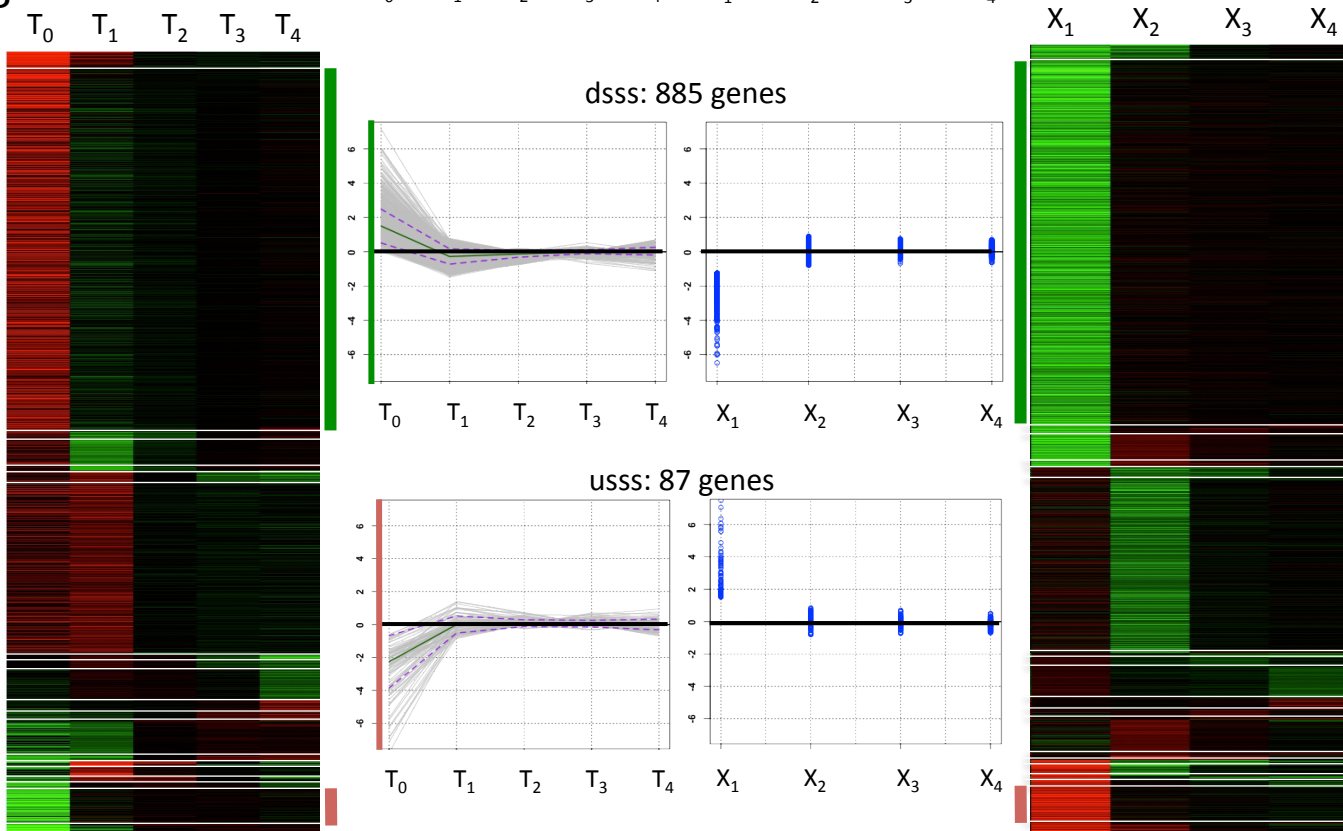

Supplement: Additional file 2: Figure S2 — Expression profile visualization of published clusters [3] and clusters obtained from discrete transition profiles. A-B: Left panels: heatmaps representing expression profiles from T0 to T4. Red, green and black indicate expression over, under or equal to the median value along the five time points. Middle left panel: temporal profiles. x-axis indicates the time points, y-axis indicates the log2 signal value, the green line corresponds to the mean signal value, the dashed purple line corresponds to the standard deviation, each grey line represents a gene in the cluster. Middle right panels: transition profiles. x-axis indicates the transitions X1 to X4 between consecutive time points, y-axis indicates the log ratio signal value, each blue circle represents a gene. A. Right panels: Schematization of expression profiles of all clusters defined by Pilot et al., the numbers over the curves indicate the number of genes.The colors of the curves correspond to the vertical line colors in the other panels. B. Right panel: heatmap representing the transition profiles from X1 to X4. Red, green and black indicate expression up-, down-regulation or stability of expression during the variations. [file 1471-2164-14-226-S2.pdf]

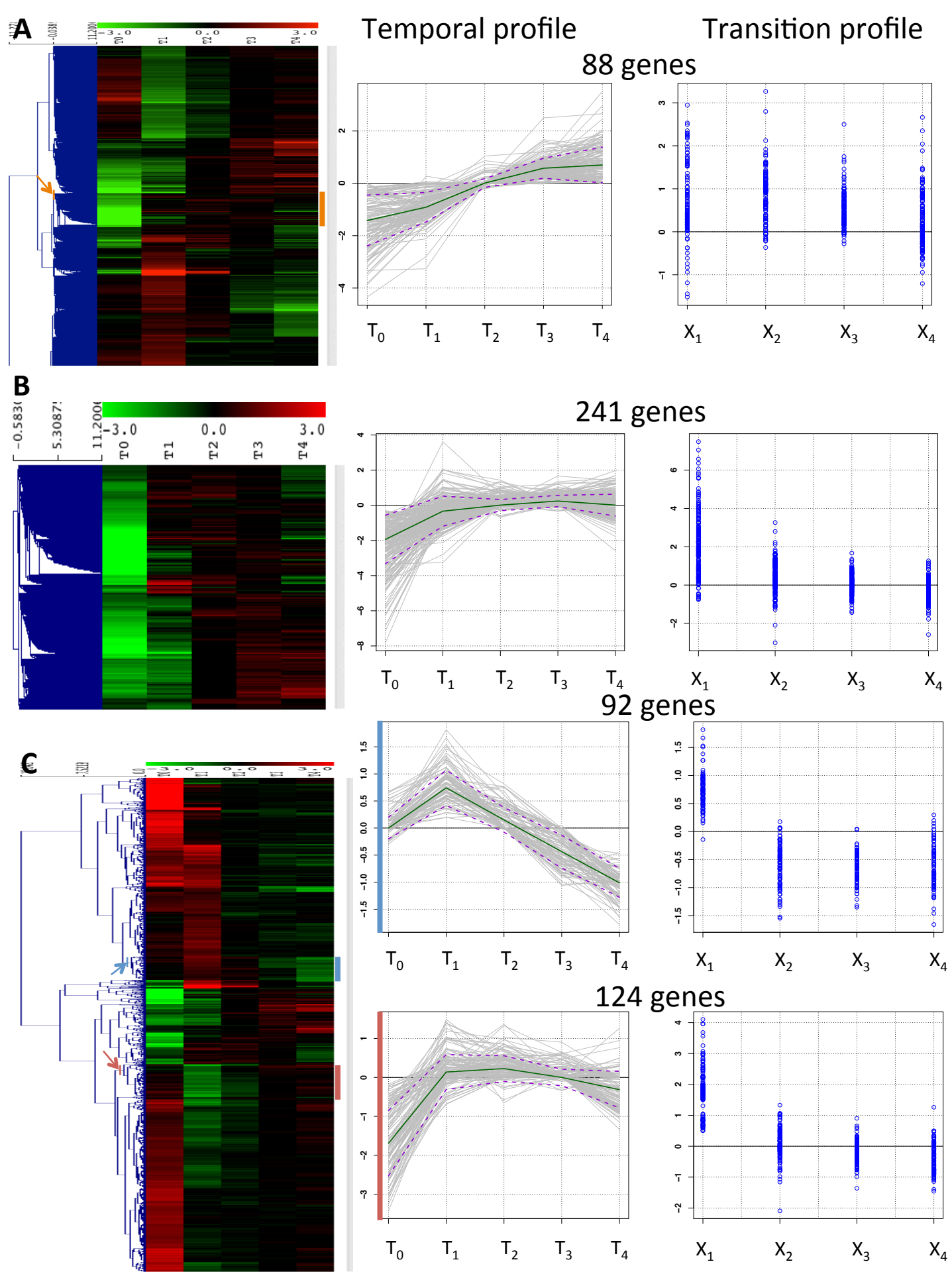

Supplement: Additional file 3: Figure S3 — Expression profile visualization of clusters obtained from Pilot at al. [3] data using classical clustering methods. Heatmap representing expression profiles from T0 to T4. Red, green and black indicate expression over, under or equal to the median value along the five time points. A. Hierarchical clustering using dot product metrics and complete linkage. B. One of the cluster obtained with K-means partitioning (a priori 10 clusters) by 50 iteration. C. Hierarchical clustering using euclidian distance and complete linkage. Middle panel: temporal profiles. x-axis indicates the time points, y-axis indicates the log2 signal value, the green line corresponds to the mean signal value, the dashed purple line corresponds to the standard deviation, each grey line represents a gene in the cluster. Right panel: transition profiles. x-axis indicates the transitions X1 to X4 between consecutive time points, y-axis indicates the log ratio signal value, each blue circle represents a gene. [file 1471-2164-14-226-S3.pdf]

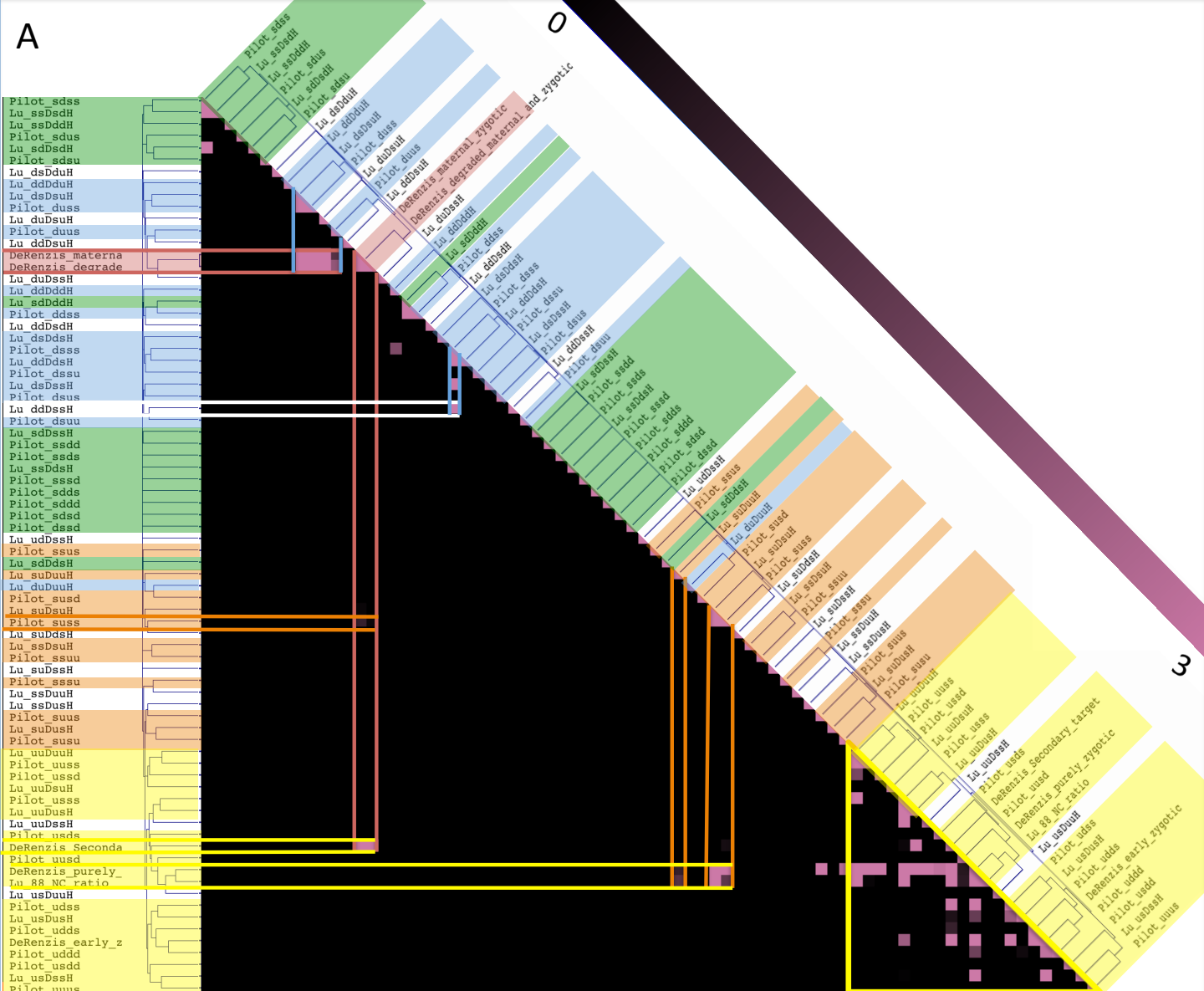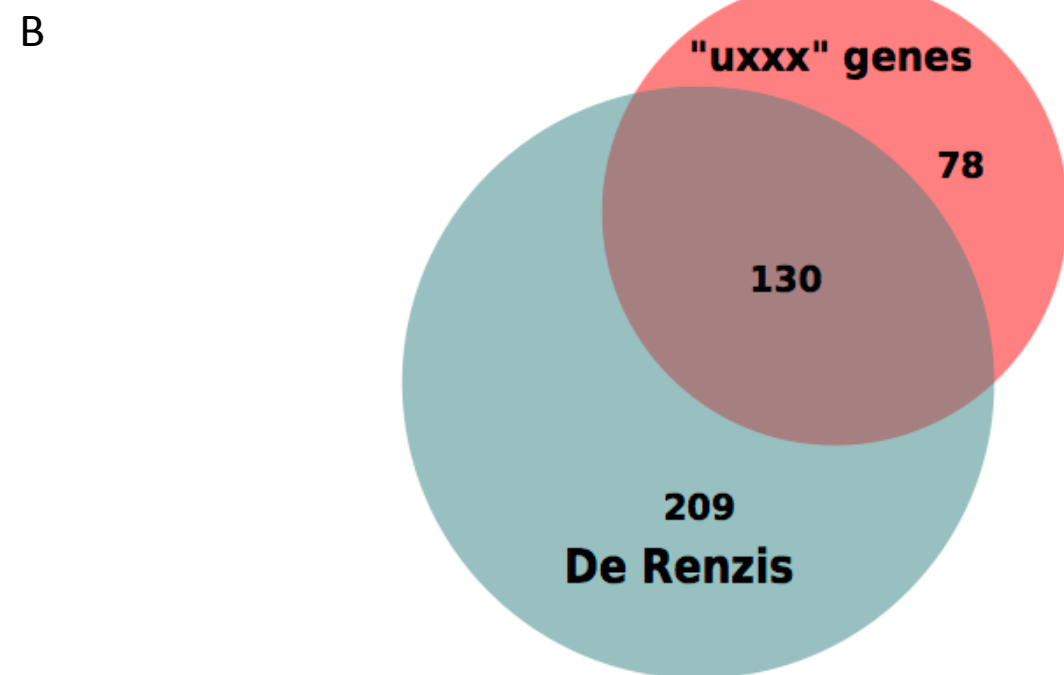

Supplement: Additional file 5: Figure S4 — (A) Gene content comparison between clusters of co-expressed genes in function of their significant overlap. Colors highlight clusters containing genes having the same expression pattern: yellow denotes genes significantly activated during the 1st and early second wave of ZGA; orange denotes genes lately activated (end of cellularisation and gastrulation); blue and green denote genes whose transcripts are maternally provided and significantly early and lately degraded, respectively; finally, red denotes clusters containing genes whose transcripts are provided both maternally and zygotically. Uncolored clusters were extracted from the data of Lu et al. and do not correspond to any known regulatory mechanisms (maternal clock, NC ratio). Lines in the heatmap highlight the significant overlapping between gene clusters. Color scale is represented by a diagonal black to purple gradient corresponding to significance from 0 to 3 (and beyond). (B) Venn diagram representing the overlapping between De Renzis et al. [2] early and purely zygotic gene published clusters (green) and the merged set of genes activated during ZGA derived from the discretization analysis (“uxxx” genes in red). This grouping forms the “ZGA cluster” containing 417 genes. [file 1471-2164-14-226-S5.pdf]

(A)

Proportion of sites found in CRER

(B)

Frequency of homotypic CRER

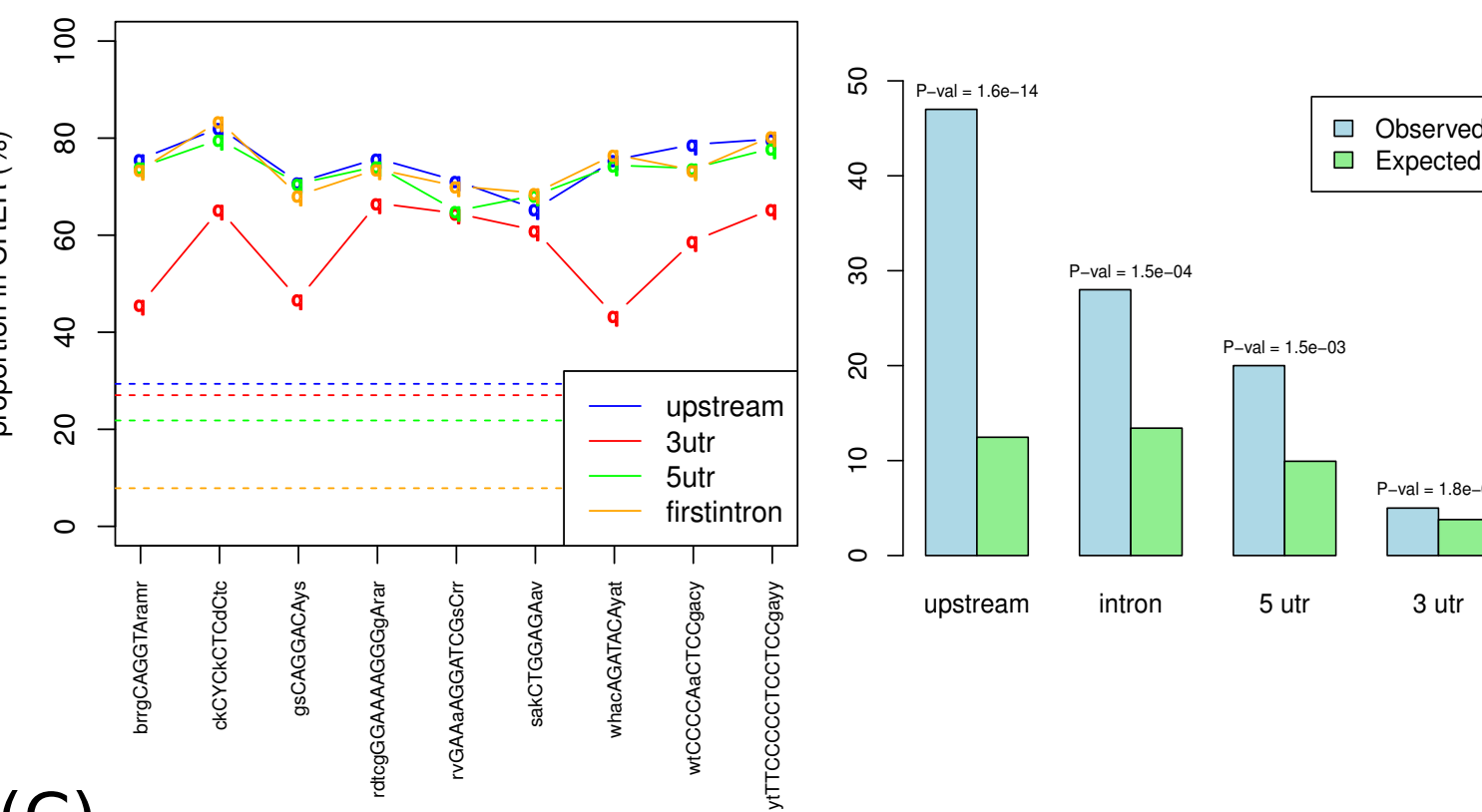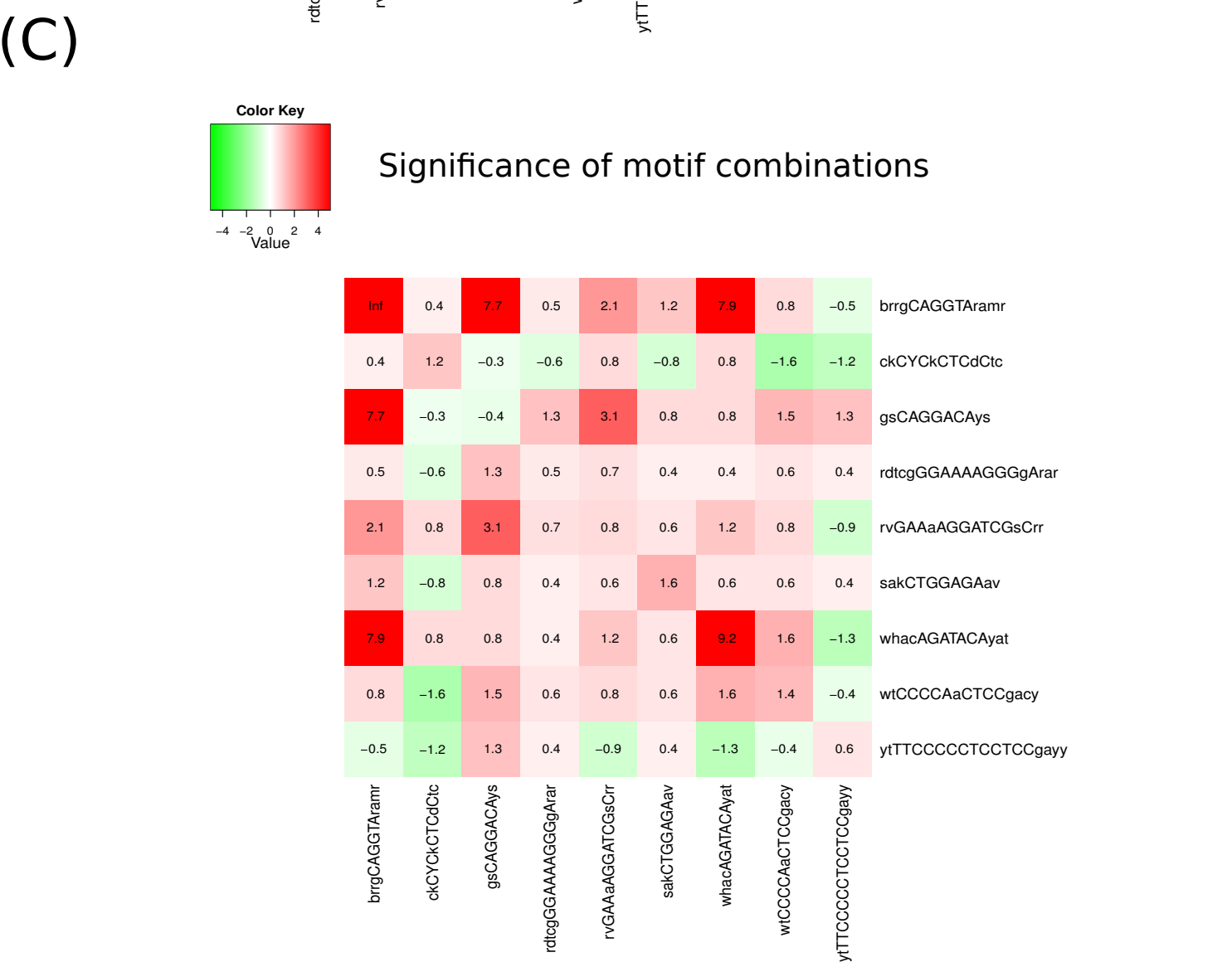

Supplement: Additional file 9: Figure S6 — Organization of CRERs. (A) Between 60% and 80% of the motif instances lie in CRERs, which represents a significant enrichment over random expectation, given that the CRER span only 15 to 30% of the regions considered (dashed lines). (B) Homotypic CRERs are found significantly more often than expected from a randomization procedure preserving overall motif frequency and CRER motif density. This enrichment is particularly pronounced in the upstream regions. Shown is the p-value based on a Poisson distribution of expected number of instances. (C) Significance of homo-/heterotypic configurations. The first three patterns correspond to the known Zelda, TRL and TTK motifs. Zelda (CAGGTA) and AGATACA motif show striking enrichment in homotypic configurations, while heterotypic configurations containing Zelda together with either TTK (CAGGACA) or AGATACA-motif are significantly more frequent than expected. Numbers indicate the significance, i.e. -log10(Q-value). [file 1471-2164-14-226-S9.pdf]

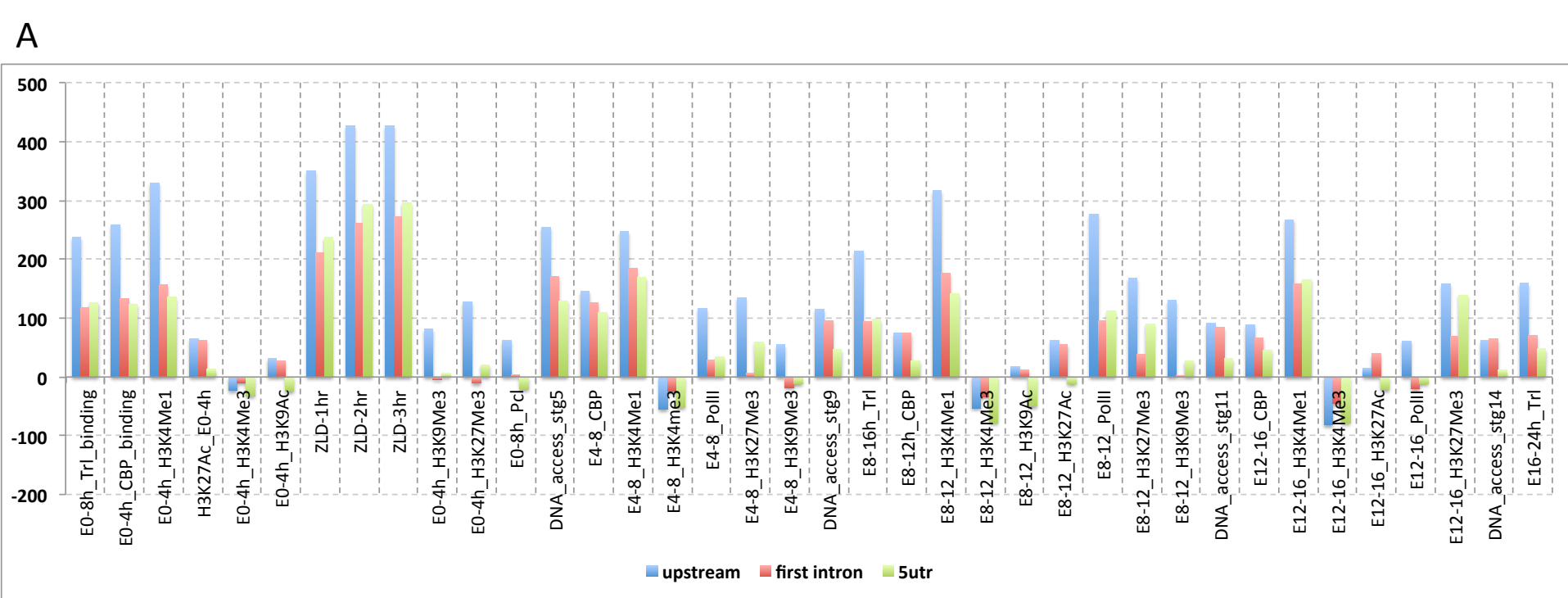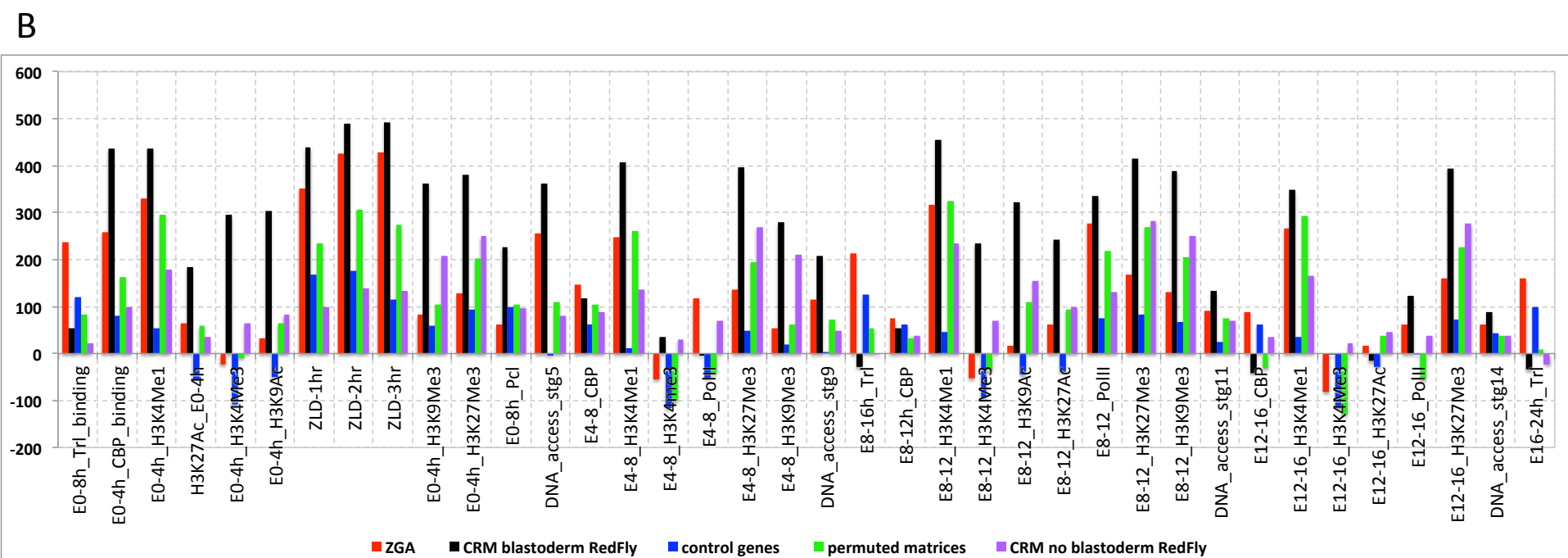

Supplement: Additional file 13: Figure S9 — AUC measuring the capability of various epigenetic marks to discriminate ZGA regions and CRM from random selections. Distribution of AUC values (ordinate) obtained from 38 genome-wise location experiments (abscissa) and predicted CRMs from different type of ZGA non-coding sequences (A) or predicted CRMs in ZGA upstream sequences, blastoderm CRMs from RedFly and negative controls (B). [file 1471-2164-14-226-S13.pdf]

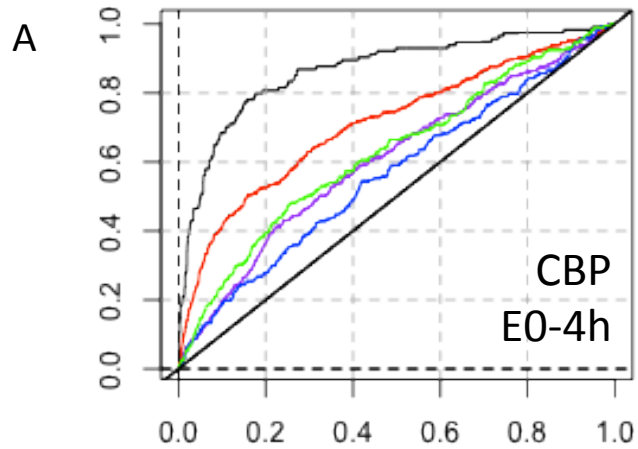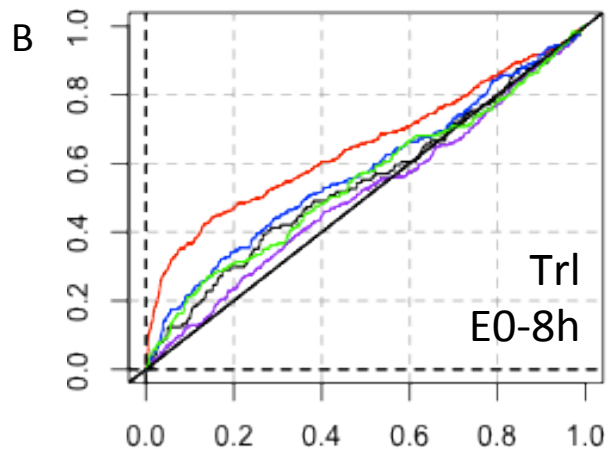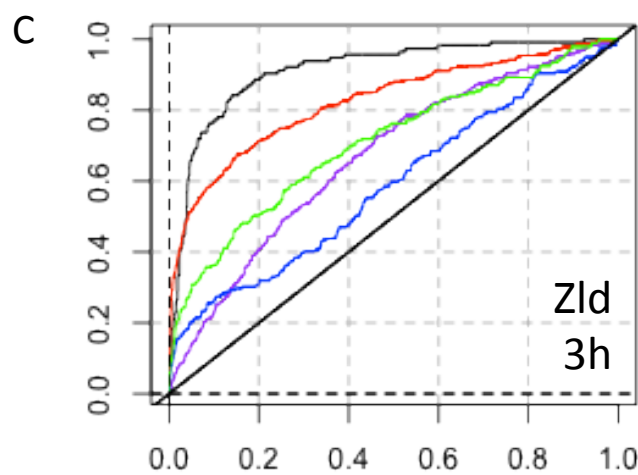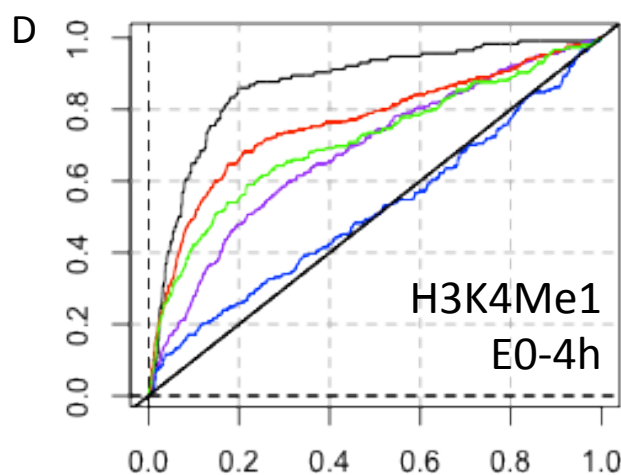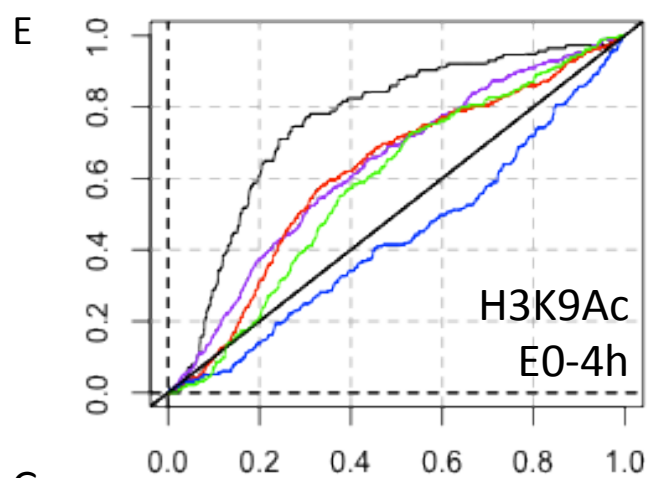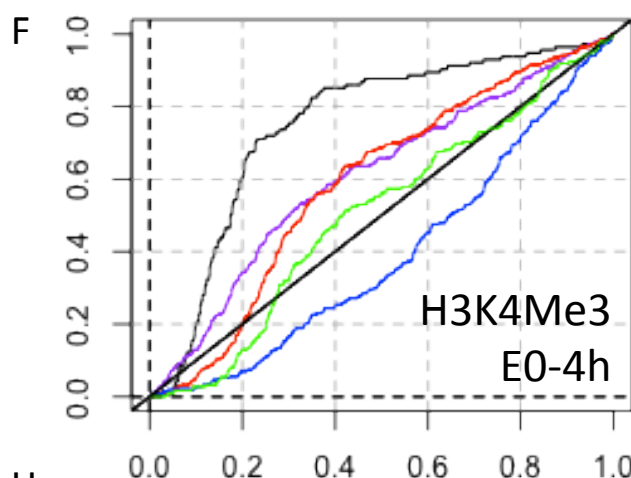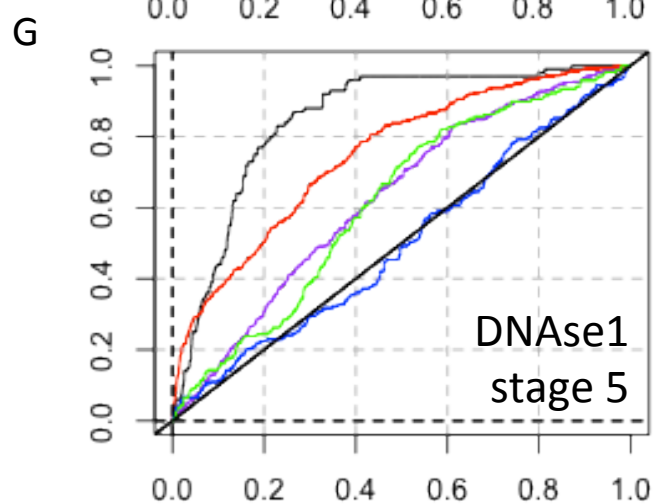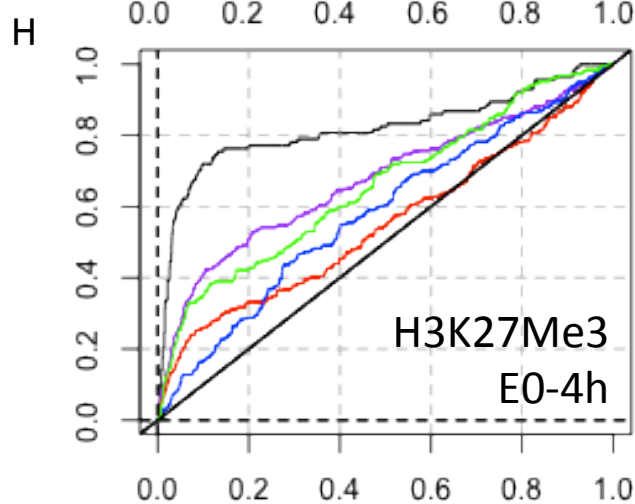

Supplement: Additional file 14: Figure S10 — ROC curves showing the enrichment in reads for various types of genomic regions (predicted CRMs, annotated CRMs, random controls). The ordinate and abscissa represent respectively the fractions of test regions (Sensitivity) and random regions (False Positive Rate) passing a given threshold of density. The kind and time window of each dataset is specified in the right corner. Different line colors denote different types of test regions. Black: 114 CRMs annotated in RedFly database as enhancing expression in the blastoderm embryo; purple: 317 CRMs supposed to be silent in early embryo, according to RedFly annotations; red: 528 CRMs predicted by scanning the 5kb upstream regions of the ZGA genes with nine discovered motifs; blue: 164 CRERs predicted by scanning the 5kb upstream regions of 417 random genes with the same matrices; green: 151 CRERs predicted by scanning the 5kb upstream regions of the ZGA genes with nine randomly column-permuted matrices. [file 1471-2164-14-226-S14.pdf]

A

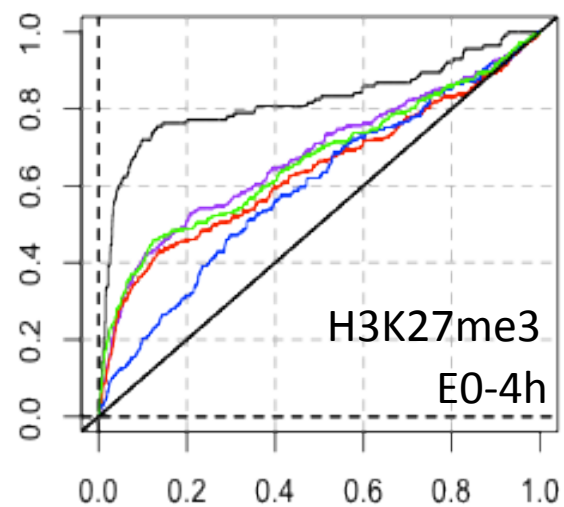

B

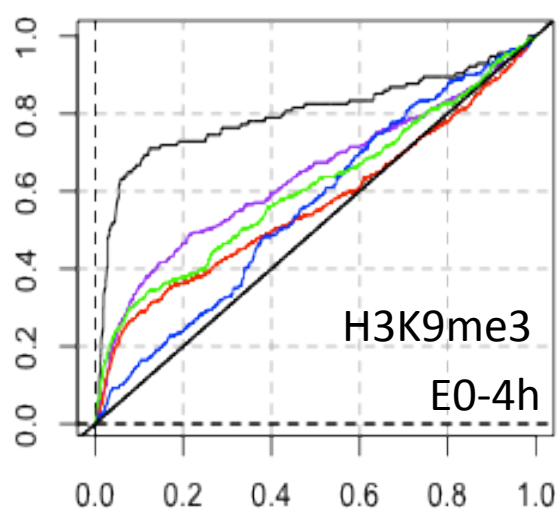

C

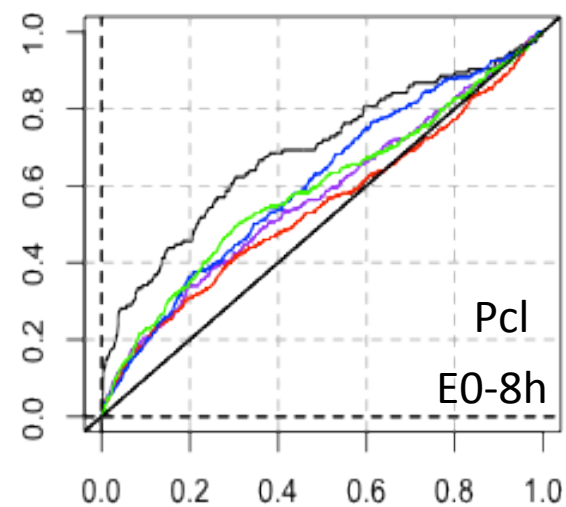

D

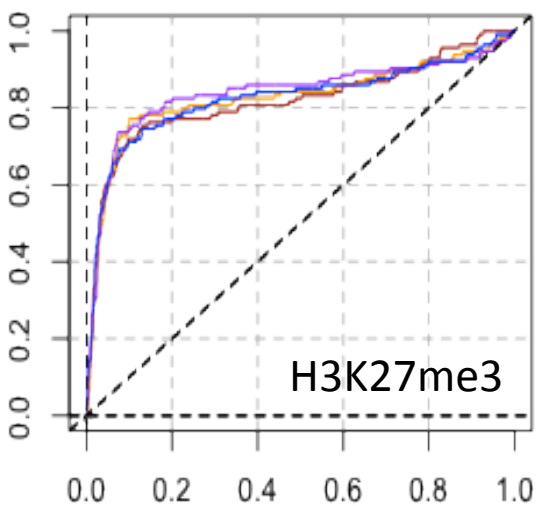

E

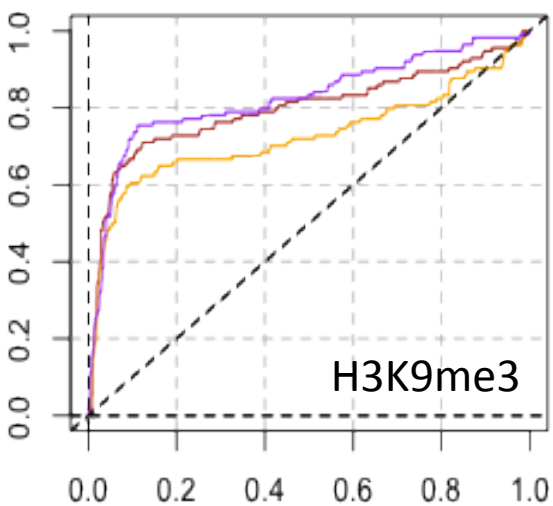

F

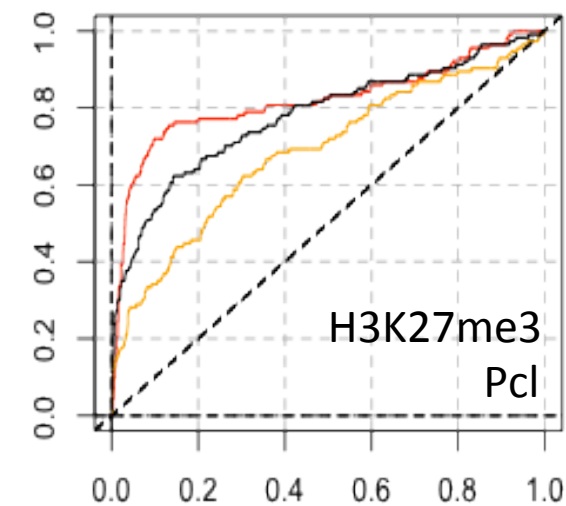

Supplement: Additional file 15: Figure S11 — ROC curves representing enrichment of CRMs for repressive marks and evolution along the development. A-C: Black: 114 CRMs annotated in RedFly database as enhancing expression in the blastoderm embryo; purple: 317 CRMs supposed to be silent in early embryo, according to RedFly annotations; red: 528 CRMs predicted by scanning the 5kb upstream regions of the ZGA genes with nine discovered motifs; blue: 164 CRERs predicted by scanning the 5kb upstream regions of 417 random genes with the same matrices; green: 151 CRERs predicted by scanning the 5kb upstream regions of the ZGA genes with nine randomly column-permuted matrices. D-F: Red, orange, purple and blue denote different timing from the earliest to the latest. [file 1471-2164-14-226-S15.pdf]
