# Supplementary material for: Transcriptional and epigenetic signatures of zygotic genome activation during early drosophila embryogenesis
Source: BMC Genomics. 2013 Apr 5;14:226. doi: 10.1186/1471-2164-14-226 (PMC3706223; doi:10.1186/1471-2164-14-226)

Motif	Enrichment score
vfl_SANGER_5_FBgn0259789	12.759135723067
CAGGTAG	11.1380836470664
PF0076.1-CAGGTA	11.0717022210421
vfl_SOLEXA_5_FBgn0259789	10.2502212575165
CASGTAR	9.74366411671557
TACCTGC	8.85525369244997
MA0340.1-MOT3	4.24295891331343
GAGAGAG	3.46645717797162
CTCTCTC	3.46645717797162
M00043-I-DL_01	3.35453118268841
dl_FlyReg_FBgn0000462	3.24723752294856
dl	3.24723752294856
RAAMGGRTTA-Kruppel	3.24664910672761
M00774-V-NFKB_Q6_01	3.19416468733339
Gsc_Cell_FBgn0010323	3.17917161127211
bcd_FlyReg_FBgn0000166	3.13986886898448
bcd	3.13986886898448
Trl_FlyReg_FBgn0013263	3.09551843852693
Trl	3.09551843852693
MA0205.1-Trl	3.09551843852693
M00723-I-GAGAFACTOR_Q6	3.04762020438423
Oc_SOLEXA_FBgn0004102	3.00892895346348
Ptx1_SOLEXA_FBgn0020912	2.98415778431874
TIFDMEM0000009	2.93688834790237
CGTGNGAA	2.93685373518348
Bcd_SOLEXA_FBgn0000166	2.93221563085364
MA0022.1-dl_1	2.92012425439175
M01111-V-RBPJK_Q4	2.9091693288664
M00412-V-AREB6_01	2.86589189269411
Bcd_Cell_FBgn0000166	2.85202949878289
AGAGAGCG	2.83936124367301
Kr_FlyReg_FBgn0001325	2.79895089438123
Kr	2.79895089438123
PF0086.1-GGGNNTTTCC	2.79666645493519
MA0306.1-GIS1	2.7539658974109
MA0372.1-RPH1	2.73781906405363
AGAGAGAG	2.73031964162975
M01112-V-RBPJK_01	2.65071615699347
Kr_NAR_FBgn0001325	2.60858670933069
M00234-I-SUH_01	2.59301098583493
MA0023.1-dl_2	2.58319251124613
Gsc_SOLEXA_FBgn0010323	2.5705069497768
M00935-V-NFAT_Q4_01	2.56416705343538
PH0035.1-Gsc	2.53477508632023

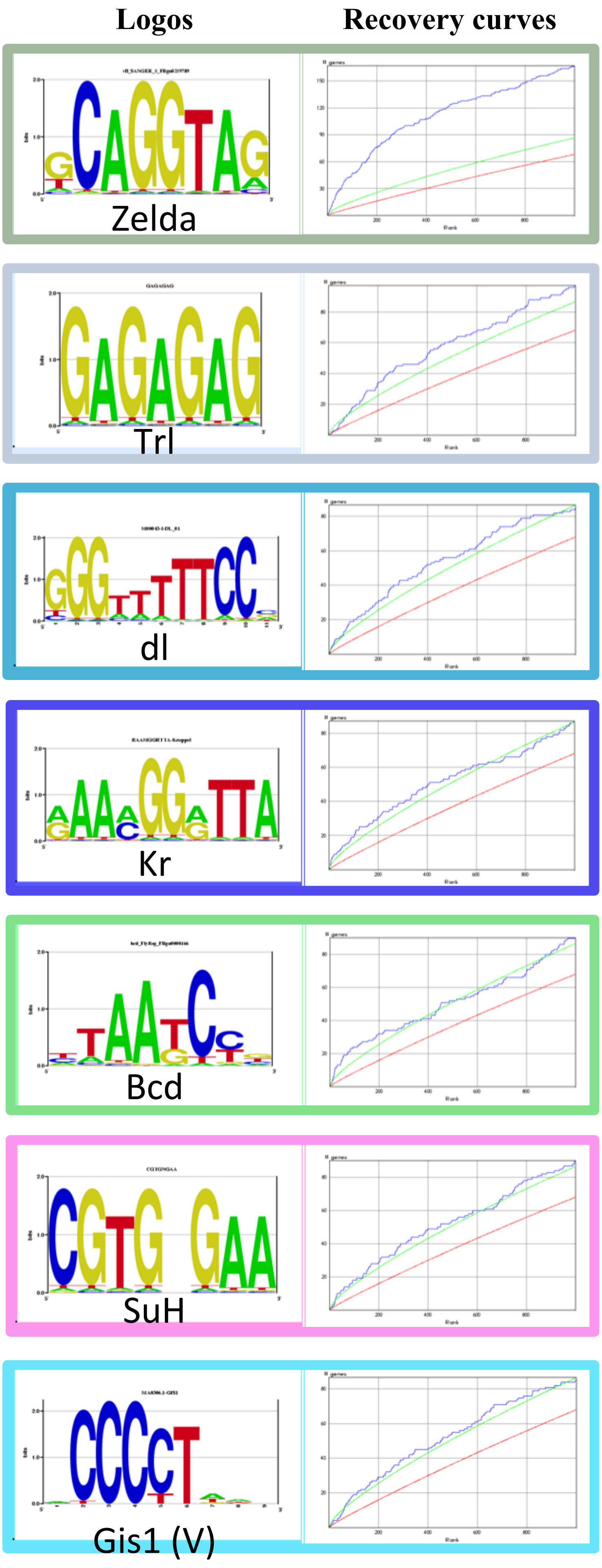

Supplement: Additional file 8: Figure S5 — (A) Summary of CisTargetX results. The blue curves of the ROC graphs represent the ranking of ZGA genes (ordinate) among all Drosophila melanogaster genes (abscissa) (see [23] for details). The red curve represents the mean of the scores for all matrices of the reference databases, and the green curve indicates a confidence interval (2 sd from the mean curve). The colors of the lines match that of the contours of the corresponding binding motifs (the use of several motif databases generates redundancy). The logo displayed corresponds to the motif with the best enrichment score within the group of similar motifs. Under each logo, the corresponding transcription factor is specified. [file 1471-2164-14-226-S8.pdf]
