# Supplementary material for: Transcriptional and epigenetic signatures of zygotic genome activation during early drosophila embryogenesis
Source: BMC Genomics. 2013 Apr 5;14:226. doi: 10.1186/1471-2164-14-226 (PMC3706223; doi:10.1186/1471-2164-14-226)
Supplement: Additional file 10: Figure S7 — peak-motifs differential analyses between ZGA and non-ZGA peaks for CBP, TRL, H3K4me1 and DNAse1 accessibility. The circle inclusions indicates the subset of peaks overlapping coding sequences of the ZGA genes (dark blue circle) relative to the total peak set (green circle). Circle surfaces are proportional to the numbers of peaks (indicated besides the circles using the same color code). This representation makes clear that the large majority of peaks fall into non coding regions. For each experiment, we indicate the number of Drosophila genes and ZGA genes containing at least one peak, and the binomial p-value of the enrichment of peaks in ZGA non-coding regions according to the expected frequency of peaks per nucleotide (all Drosophila non-coding sequences). The last column summarizes the results (logo of over-represented motifs, their significance and the percentage of peaks carrying at least on motif occurrence) of the differential analysis performed with peak-motifs between ZGA versus non ZGA peaks. [file 1471-2164-14-226-S10.pdf]

<table>
<tr><th rowspan="2"></th><th rowspan="2"></th><th rowspan="2">Nb of gene containing at least one peak (total:61.3 Mb)</th><th rowspan="2">Nb ZGA gene containing at least one peak (total:3Mb)</th><th rowspan="2">Binomial p.value</th><th colspan="3">Differential analysis ZGA vs non-ZGA peaks</th></tr>
<tr><th>Logos</th><th>sig</th><th>peaks with site</th></tr>
<tr><td rowspan="2">CBP E0-4h</td><td rowspan="2">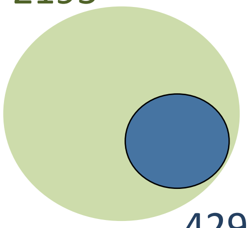</td><td rowspan="2">2056</td><td rowspan="2">237</td><td rowspan="2">2.3e-118</td><td>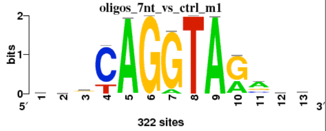</td><td>11.8</td><td>42.66%</td></tr>
<tr><td>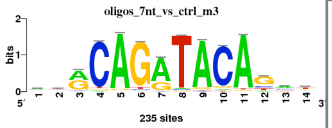</td><td>6.6</td><td>29.37%</td></tr>
<tr><td rowspan="2">Trl E0-8h</td><td rowspan="2">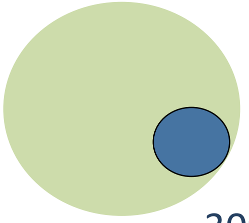</td><td rowspan="2">2329</td><td rowspan="2">189</td><td rowspan="2">1e-31</td><td>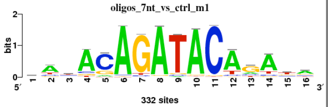</td><td>9.69</td><td>59.55%</td></tr>
<tr><td>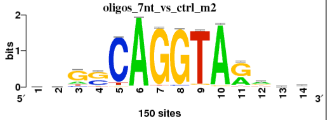</td><td>7.21</td><td>28.16%</td></tr>
<tr><td>H3K4me1 E0-8h</td><td>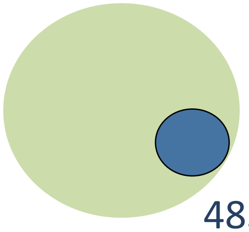</td><td>4577</td><td>239</td><td>1.6e-41</td><td>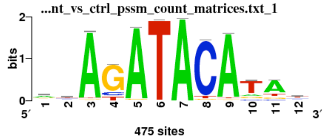</td><td>11.18</td><td>47.62%</td></tr>
<tr><td rowspan="3">DNAse1 stage 5</td><td rowspan="3">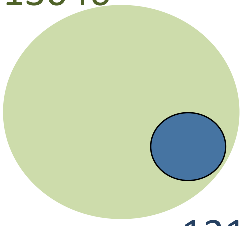</td><td rowspan="3">8579</td><td rowspan="3">352</td><td rowspan="3">3.1e-117</td><td>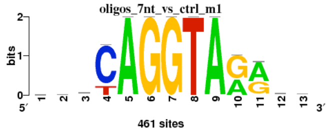</td><td>60.67</td><td>26.56%</td></tr>
<tr><td>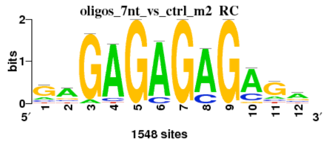</td><td>40.56</td><td>26.48%</td></tr>
<tr><td>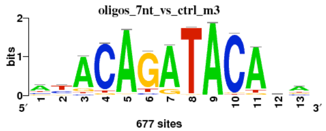</td><td>26.63</td><td>36?27%</td></tr>
</table>
