# Supplementary material for: Transcriptional and epigenetic signatures of zygotic genome activation during early drosophila embryogenesis
Source: BMC Genomics. 2013 Apr 5;14:226. doi: 10.1186/1471-2164-14-226 (PMC3706223; doi:10.1186/1471-2164-14-226)
Supplement: Additional file 11: Figure S8 — peak-motifs differential analyses between ZGA and non-ZGA peaks for Zelda. Confer to Additional file 10: Figure S7 legend. [file 1471-2164-14-226-S11.pdf]

		Nb of gene containing at least one peak (total:61.3 Mb)	Nb ZGA gene containing at least one peak (total:3Mb)	Binomial p.value	Differential analysis ZGA vs non-ZGA peaks: Logos	sig	peaks with site
Zelda 1h	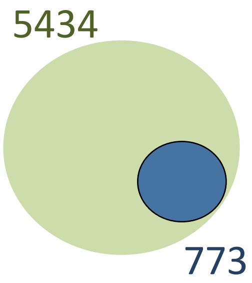	3420	266	8.6e-137	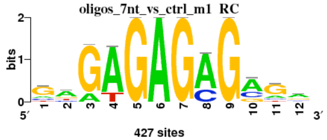	9.97	15.39%
					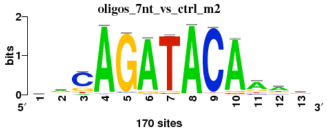	7.20	15.91%
Zelda 2h	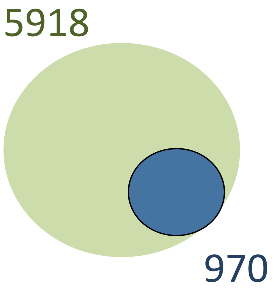	3247	295	2.3e-211	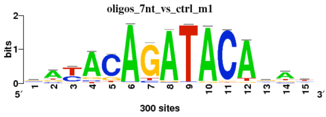	10.7	23.92%
					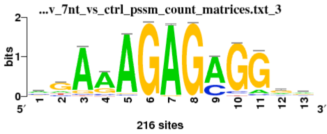	3.36	21.65%
Zelda 3h	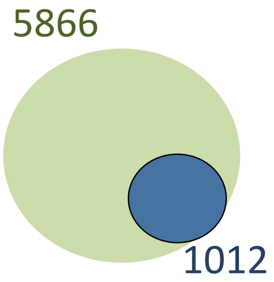	3144	298	2.8e-236	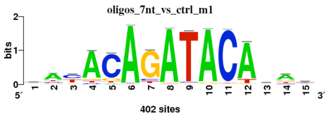	7.65	28.75%
