# Supplementary material for: Transcriptional and epigenetic signatures of zygotic genome activation during early drosophila embryogenesis
Source: BMC Genomics. 2013 Apr 5;14:226. doi: 10.1186/1471-2164-14-226 (PMC3706223; doi:10.1186/1471-2164-14-226)

A

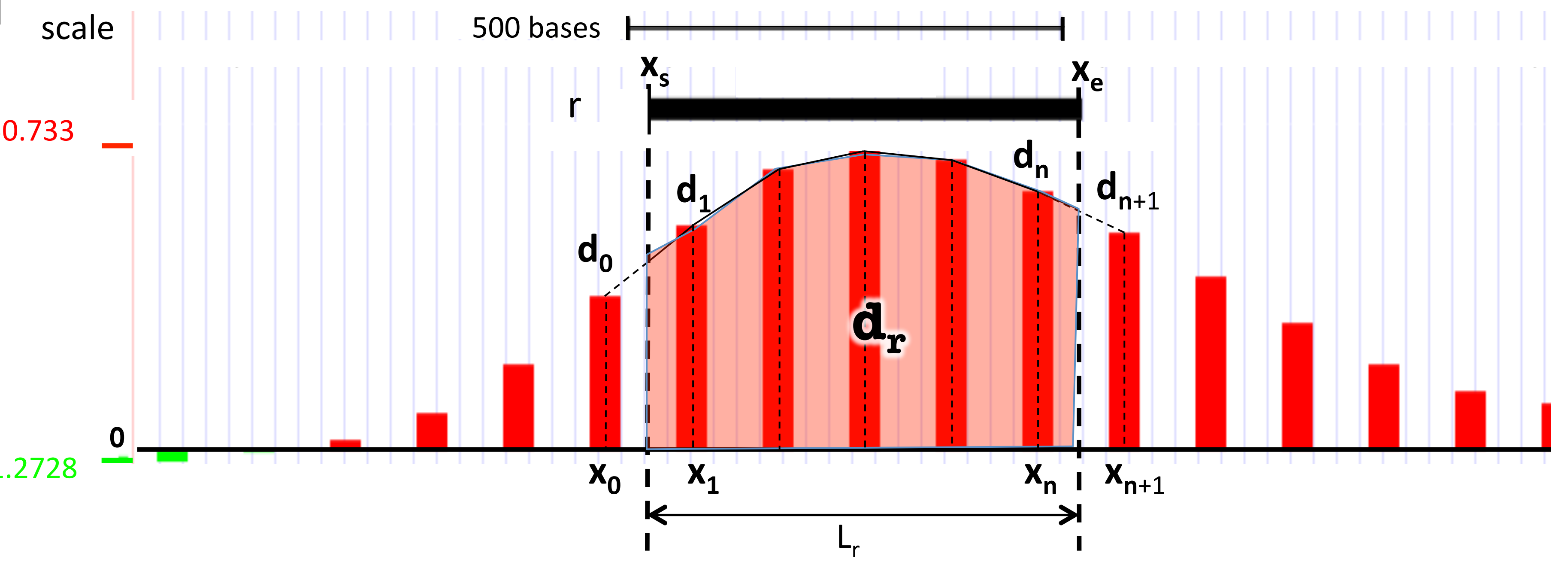

B

chromosome	start	end	D_R	region type	rank
chr2R	16426864	16426902	2334,47	test	1
chr2R	17592183	17592223	1096,54	test	2
chr2L	18968823	18968855	1042,04	random	3
chr2R	3849310	3849356	886,96	test	4
chr3L	2197420	2197451	870,83	random	5
chr3L	2483107	2483185	768,65	test	6
chr2L	8510480	8510514	745,23	random	7
chr2L	8214269	8214301	708,17	random	8
chr3R	1498044	1498083	700,58	test	9
chr2R	13280079	13280149	663,71	random	10

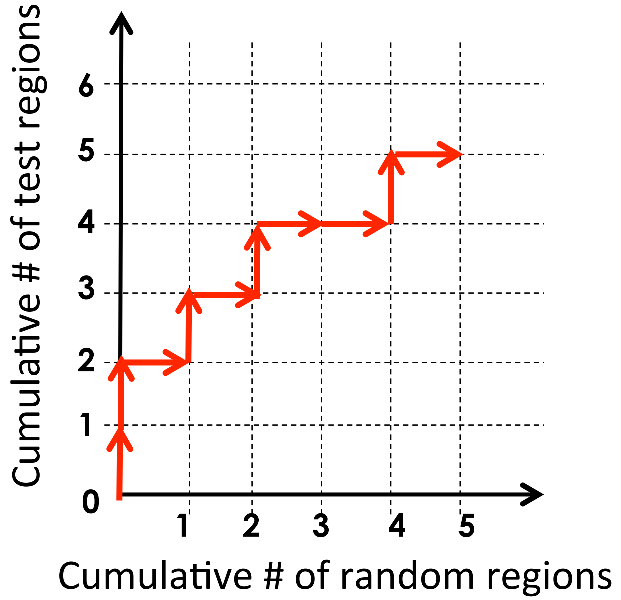

Supplement: Additional file 16: Figure S12 — Principle of the analysis of region enrichment in reads. (A) Illustration of values used for the computation of the intensity under a given region Ir as defined in Methods section. The histogram represents the read density tracks. Green read density tracks represent negative values (the lowest is indicated in the margin in green), while red read density tracks represent positives ones (the highest is indicated in the margin in red). The black curve represents the linear extrapolation of read density under the region. (B) The table lists the genomic positions (chromosome, start, end), Ir, the type (random or regions to test) and the corresponding rank for each region considered. The ROC curve (right) displays the cumulative numbers of random (abscissa) and test (ordinate) regions found according to their ranking. [file 1471-2164-14-226-S16.pdf]
